# Explaining Observed Infection and Antibody Age-Profiles in Populations with Urogenital Schistosomiasis

**DOI:** 10.1371/journal.pcbi.1002237

**Published:** 2011-10-20

**Authors:** Kate M. Mitchell, Francisca Mutapi, Nicholas J. Savill, Mark E. J. Woolhouse

**Affiliations:** Centre for Immunology, Infection and Evolution, Institute of Immunology & Infection Research, School of Biological Sciences, University of Edinburgh, Edinburgh, United Kingdom; Emory University, United States of America

## Abstract

Urogenital schistosomiasis is a tropical disease infecting more than 100 million people in sub-Saharan Africa. Individuals in endemic areas endure repeated infections with long-lived schistosome worms, and also encounter larval and egg stages of the life cycle. Protective immunity against infection develops slowly with age. Distinctive age-related patterns of infection and specific antibody responses are seen in endemic areas, including an infection ‘peak shift’ and a switch in the antibody types produced. Deterministic models describing changing levels of infection and antibody with age in homogeneously exposed populations were developed to identify the key mechanisms underlying the antibody switch, and to test two theories for the slow development of protective immunity: that (i) exposure to dying (long-lived) worms, or (ii) experience of a threshold level of antigen, is necessary to stimulate protective antibody. Different model structures were explored, including alternative stages of the life cycle as the main antigenic source and the principal target of protective antibody, different worm survival distributions, antigen thresholds and immune cross-regulation. Models were identified which could reproduce patterns of infection and antibody consistent with field data. Models with dying worms as the main source of protective antigen could reproduce all of these patterns, but so could some models with other continually-encountered life stages acting as the principal antigen source. An antigen threshold enhanced the ability of the model to replicate these patterns, but was not essential for it to do so. Models including either non-exponential worm survival or cross-regulation were more likely to be able to reproduce field patterns, but neither of these was absolutely required. The combination of life cycle stage stimulating, and targeted by, antibody was found to be critical in determining whether models could successfully reproduce patterns in the data, and a number of combinations were excluded as being inconsistent with field data.

## Author Summary

Urogenital schistosomiasis is a tropical infectious disease caused by schistosome blood flukes, infecting more than 100 million people in sub-Saharan Africa. Protective immunity develops against schistosomes, but takes many years to do so, with children acquiring multiple infections with long-lived parasites. It is important to understand how this protection develops, both to aid vaccine development, and to understand the long-term implications of other control strategies which may reduce or alter natural exposure to the parasite. We used mathematical models to investigate possible explanations for this slow development of protection, including the possibility that when worms die (after surviving in the host for 3–10 years) they release large quantities of the molecules needed to stimulate a protective immune response, or the idea that an individual's infection burden needs to exceed a threshold number of parasites before they mount a protective immune response. We found that these mechanisms were consistent with patterns of levels of infection and antibody measured in populations with schistosome infection, but that neither was essential to explain these patterns. We found that the combination of the stage of the parasite life cycle which stimulated protective immunity, and the life cycle stage targeted by this immunity, was critical.

## Introduction

Urogenital schistosomiasis, which is caused by the blood fluke *Schistosoma haematobium*, is a tropical disease of great public health importance, infecting more than 100 million people in sub-Saharan Africa [1], [2]. *S. haematobium* has a complex lifecycle, which includes free-living stages in the environment and asexual reproduction in intermediate freshwater snail hosts, as well as maturation and sexual reproduction within mammalian hosts [1], [3]. Humans acquire infection through contact with water bodies containing the infective stages (cercariae). Humans are exposed to the larval (cercariae and schistosomulae), adult worm and egg stages of the schistosome life cycle. The number of adult worms infecting a person cannot be measured directly, but is estimated from egg output in urine [4], and the average life span of the adult worm is estimated to be around 3–10 years [5], [6].

Infection intensities in populations with endemic urogenital schistosomiasis consistently follow a ‘peaked’ (or convex) curve with age, with infection levels peaking between the ages of 6–20 years old, and lower infection intensities found in older adults [7], [8], [9]. In areas with higher overall levels of infection, the peak in infection intensity tends to be higher and occurs at an earlier age than in areas with lower infection levels. This pattern is described as a peak shift [10]. Previous modelling work has demonstrated that the peak shift is consistent with the development of acquired protective immunity, as a function of cumulative exposure to schistosome antigens [11]. Age-related changes in the nature of the immune response generated against schistosomes have also been reported. Two studies in Zimbabwe have identified different groups of schistosome-specific antibody sub-classes (isotypes) which display contrasting age profiles. One group of isotypes was shown to rise with host age while a second group declined in older individuals [12], [13]. Both studies reported negative correlations between antibodies from the two different groups. The ‘switch’ between the different antibody responses occurred after the peak in infection intensity for both populations. Some of the isotypes which increased with age in these populations have been associated with protection against re-infection in other studies, particularly IgE specific for adult worm antigen preparations [14], but these protective responses tend to develop slowly with age, despite frequent exposure to infection from an early age [15].

In this study, two hypotheses were explored for the slow development of protective immunity. The first hypothesis was that dying worms are the main source of protective antigen. It has been observed that treatment with the antihelminthic drug praziquantel, which kills adult worms, induces an antibody switch in young children similar to the switch seen occurring naturally in older children [16]. It is hypothesised that the long life span of schistosome worms delays exposure to protective antigens, delaying the development of natural protective immunity [17]. Second, it has been proposed that exposure to a certain threshold level of antigen is needed before protective responses can be stimulated [17], [18]. This is supported by the finding that, in a *S. haematobium* endemic area, older or more heavily infected groups produced detectable antibody against a wider range of schistosome antigens [18].

Mathematical models of schistosome and other helminth infections have previously been used to identify mechanisms capable of explaining the peaked age-intensity curve and the peak shift [11], [19], [20]. Such patterns can often be reproduced by several different models which imply different underlying mechanisms [21], [22]. Pattern-oriented modelling (POM), an approach developed in ecological modelling, can be used to identify models which simultaneously replicate multiple different patterns observed in real systems at different levels or scales [23]. This approach enables quantitative or qualitative patterns to be used, and the requirement for models to replicate multiple different patterns can greatly increase their discriminatory power. POM has been used in ecological modelling to distinguish between possible model structures, for example to choose between different models for tree-thinning which made different assumptions about the distribution of initial plant size and growth rates, and were compared for their ability to give realistic levels of plant size inequality and canopy diameter, as well as reproducing the well-established relationship between plant density and plant size [24]. POM has also been used in ecological modelling to reduce parameter uncertainty, for example in models of amphibian population dynamics, where there was greater confidence in the model structure, but certain key parameters (juvenile and adult toad survival) were unknown [25]. A similar approach has also been used to infer pre-breeding survival rates for woodpeckers [26].

This study used a pattern-oriented modelling approach to determine whether previously proposed epidemiological and immunological mechanisms were able to explain all of the key patterns in infection and antibody observed in field studies of *S. haematobium*. Previous modelling work has generally focussed upon reproducing patterns of infection intensity rather than antibody responses, so it was of particular interest whether models could reproduce antibody patterns, particularly the antibody switch. Robust patterns in infection and antibody data for *S. haematobium* endemic populations were identified and quantified to draw up model criteria. Deterministic models were used which described changing levels of infection and two separate antibody responses with age in a homogeneously exposed population. Whilst such models do not capture all of the complexity and variability in this system, they should be sufficient to capture the patterns of interest: similar models have been used previously to identify factors leading to the peaked age-intensity curve and the peak shift [10], and are expected to be able to identify the mechanisms underlying the antibody switch. The models were used to test whether one or more of the following factors were necessary to reproduce these patterns: dying worms as the principal antigen (versus other life cycle stages), non-exponential worm survival distribution (expected to affect the timing of exposure to dying worms), an antigen threshold, or cross-regulation between the two antibody responses. Each stage of the schistosome life cycle was considered as a potential source for protective antigen, since different immune responses have been shown to be stimulated by each of the different stages, which have different life spans and distinct patterns of exposure with age. In animal models, cercariae stimulate local and systemic inflammatory responses [27], whilst eggs are potent inducers of Th2 responses [28]. Despite their relatively long life span, live adult worms are associated with less potent immune stimulation, which may be associated with reduced surface antigen expression and adsorption of host molecules [29], [30]. Cross-regulation of antibody responses was also considered specifically to see whether this was necessary to generate the antibody ‘switch’ pattern, in line with the antagonism known to exist between different cytokine responses, which are involved in determining which antibody isotypes are made [31], [32]. It was found that each of these factors enhanced the ability of the models to reproduce the required patterns, but none of them were necessary for the models to be able to reproduce patterns of infection and antibody consistent with field data. The combination of the stage of the schistosome life cycle which stimulated each antibody response, and the stage of the life cycle targeted by the antibodies, was found to be informative.

## Methods

### Drawing up model criteria

Key patterns of infection and antibody seen in field data were characterized and quantified where possible, and used to draw up criteria which must all be met by a successful model. The following patterns were included: a peaked age intensity curve with control of infection in adults, a peak shift, reasonable mean worm life span and an antibody switch, occurring after the age of peak infection intensity.

Previous models have qualitatively reproduced the peaked age-intensity curve [11], [21], but here we wished to do this within limits drawn from field data. Age-intensity curves from a variety of endemic settings were used to determine limits for two criteria: the age range over which the peak in infection intensity occurs for *S. haematobium*, and the extent to which infection is reduced in adults relative to the peak level seen. The peak shift, which has previously been demonstrated for infection curves for *S. mansoni* in Kenya [21] and *S. haematobium* in Zimbabwe [33], and has also been reproduced by previous models [11], [21], was used as a qualitative criterion. Considerable variability in field data makes it difficult to put formal quantitative bounds on the peak shift, and also means that such a shift may not always be seen between any two particular study sites (although it is a strong pattern across multiple populations). The model criterion requires a shift to consistently be seen between parameter sets which differ only in their transmission rate, since the deterministic models used here do not incorporate many of the sources of variability which are likely to obscure this relationship in the data.

Previous modelling work has shown that in models with immune responses which kill adult worms, short worm life spans are predicted in older infected individuals [20]. In order to avoid accepting models which predict an unrealistically short worm life span, a criterion determining the minimum average worm life span of worms in the oldest adults in the population was set (the oldest individuals were used as worm lifespan is most likely to have settled at an equilibrium level by this age). Studies which estimate the worm life span for *S. haematobium* and *S. mansoni* in populations in which transmission has been stopped give life span estimates of 3–10 years [6], [34], [35]. A conservative cut-off of 1 year was used in the criterion to allow for uncertainties in the true duration of worm life span (bearing in mind that field estimates came from people of a variety of ages, in whom immune pressure may have been waning as a result of transmission having stopped).

The antibody switch has previously been characterised for schistosome-specific IgA and IgG1 responses in two neighbouring populations [16]. Here, we further examined data from these and other populations for which age-related *S. haematobium*-specific antibody data was available to determine how widespread the antibody switch was across different antibody isotypes and different settings, and to identify a criterion to test for the switch. Since initial model simulations indicated that cross-regulation could lead to an antibody switch being predicted in very young individuals, the age of the antibody switch relative to the age of peak infection intensity was also examined in these studies, and an additional criterion restricting the age of the antibody switch was drawn up.

#### Data sets and analysis for the criteria

Previously published data on S. haematobium infection intensity was taken from (pre-treatment) baseline studies of six Zimbabwean populations from three different field studies: Valhalla and Kaswa were both part of the Burma Valley study conducted in 1994 [12]; the Mutoko-Rusike study was conducted in 2003 [36], [37], and Magaya, Chipinda and Chitate were all part of the Murehwa study conducted in 2008/2009 [38]. Antibody data came from the Burma Valley and Mutoko-Rusike studies. Infection and antibody data were grouped into five age groups chosen to give roughly equal numbers of individuals in each group for the Burma Valley populations (age groups: ≤8, 9–10, 11–12, 13–23, 24–34 years old). Arithmetic means were calculated for infection intensities in each age group. The 24–34 year olds were used as the adult group for comparison with peak infection levels. To test for an antibody switch, significant changes in each antibody isotype with age were assessed using a Kruskal Wallis test, with age as a categorical variable, and correlations between antibody levels across the whole population were calculated using a two-tailed Spearman's rank correlation coefficient (non- parametric methods were used since the data are highly skewed). All tests used a significance cut-off level of 1%. Statistical analyses were performed using SPSS version 15. A survey of the wider literature was undertaken to identify studies which reported infection intensity levels by age, to help with the estimates for the age of peak infection intensity and relationship between adult and peak infection levels. To be included, studies had to measure infection intensities of endemic S. haematobium, had to include both children and adult subjects (over the age of 20), and had to give infection levels for at least four different age groups. Data was taken from results tables where given, otherwise it was extracted from graphs using the software application Datathief III version 1.5. A similar search for studies reporting S. haematobium-specific antibodies by age found only one suitable publication [13].

### Water contact data

Data on water contact for the Burma Valley area, which had been collected by direct observation of water contact sites, was taken from figure 1 in Chan et al. (2000) [39] using Datathief software. A two-part linear function was fitted to this data by least squares, using the solver function in Microsoft Excel 2003, with the initial part of the function constrained to pass through (0,0).

**Figure 1:**
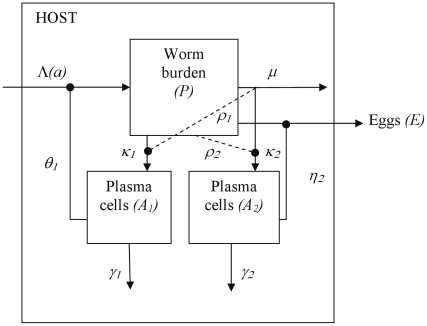
Schematic diagram of the plasma cell model. This shows the main state variables, worm burden (*P*) and two populations of plasma cells (*A*_1_ and *A*_2_), with production of eggs (*E*). A single worm compartment (*n* = 1), corresponding to exponential worm survival, is shown for clarity. Worms accumulate at an age-dependent rate Λ and die at a per-capita rate *μ*. Egg output (E) is assumed to be directly proportional to current worm burden unless anti-fecundity responses are operating. The first antibody response shown here (*A*_1_) receives its antigenic stimulus from the live worm population, and the second (*A*_2_) is stimulated by dying worms, but each response could be stimulated by any one of cercariae, live worms, dying worms or eggs. In the figure, the first antibody response (*A*_1_) is shown reducing re-infection, with relative strength *θ*_1_, and the second antibody response (*A*_2_) is reducing worm fecundity with relative strength *η*_2_, but each response could target any one of re-infection, worm death or fecundity (immune-mediated worm death is shown in figure 2). The two plasma cell populations (*A*_1_ and *A*_2_) decay at a per-cell rate of *γ_1_* and *γ_2_* respectively. Cross-regulation between the two responses (of strength *ρ*_1_ and *ρ*_2_) is shown using dashed lines. All parameters are defined in table 1 with parameter values given.

### Mathematical model

Age-related development of schistosome infection and antibody responses were modelled using a set of differential equations, using a similar framework to earlier models of helminth immunity [11], [20], [40] and immune memory development [41], [42]. Two different structures for the immune response were explored: one with only plasma cells and the other including both plasma cell and memory B cell populations. Different stages of the worm life cycle were allowed to provide the main antigenic stimulus for, and different stages of the life cycle were assumed to be the principle target of, each protective antibody response. The model with plasma cells only is presented first, followed by the memory model and the models including antigen thresholds. The equations are given below, and the model is represented schematically in figures 1–3. Parameters are defined, and parameter ranges given, in table 1.

**Figure 2 pcbi-1002237-g002:**
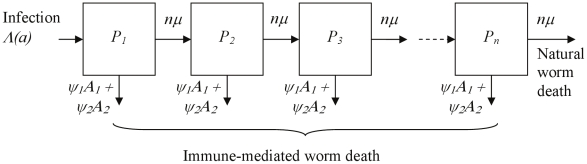
Schematic diagram of the multi-compartment worm model, used to alter the natural worm survival curve. The first three and the final (*n* ^th^) compartments are shown for a model with multiple worm compartments, with worms moving between these compartments at a constant per-worm rate, *nμ*, dying as they leave the final compartment. When either antibody response increases the rate at which worms die, worms additionally leave each compartment at a rate (*ψ*_1_ *A*_1_+*ψ*_2_ *A*_2_), where *A*_1_ and *A*_2_ are the sizes of the two plasma cell populations and *ψ*_1_ and *ψ*_2_ give the strength of immune-mediated worm killing for each antibody response. All parameters are defined in table 1 with parameter values given.

**Figure 3:**
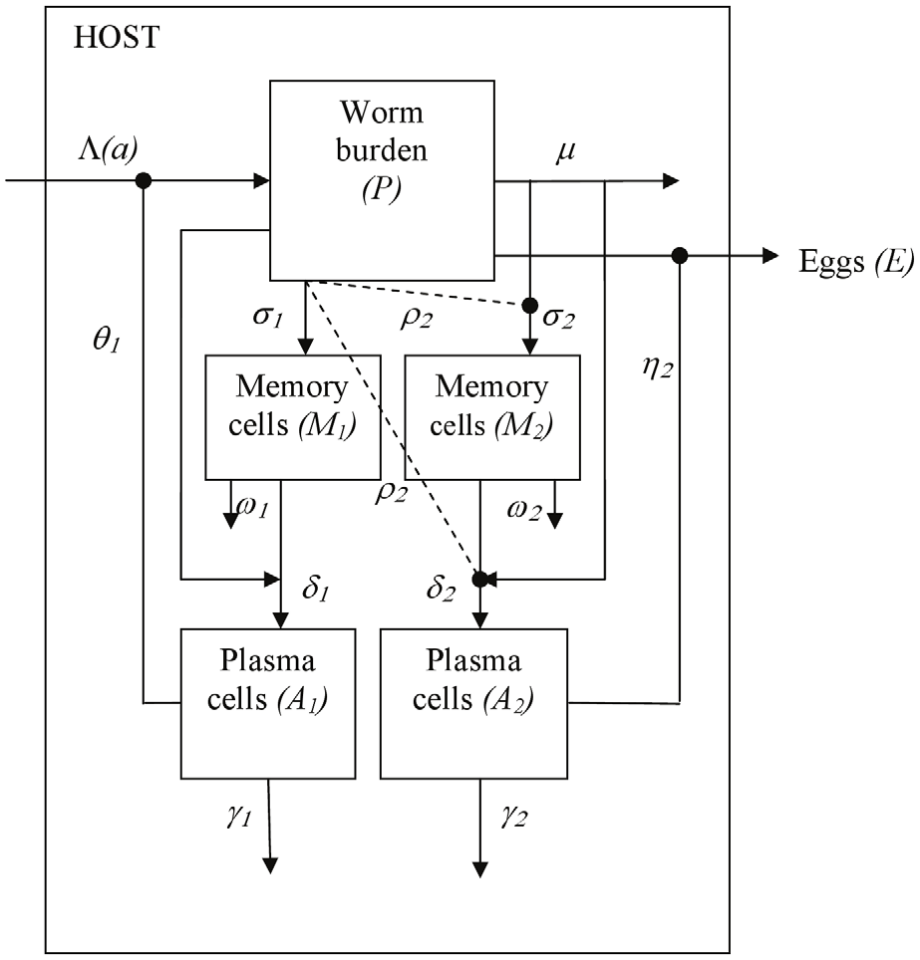
Schematic diagram of the memory model. This model has state variables for worm burden (*P*), two populations of memory cells (*M*_1_ and *M*_2_) and two corresponding populations of plasma cells (*A*_1_ and *A*_2_). Worms accumulate at an age-dependent rate Λ and die at a per-capita rate *μ*. Egg output (E) is assumed to be directly proportional to current worm burden unless anti-fecundity responses are operating. The first memory response (with associated antibody) shown here (*M*_1_ and *A*_1_) receives its antigenic stimulus from the live worm population, and the second (*M*_2_ and *A*_2_) is stimulated by dying worms, but each response could be stimulated by any one of cercariae, live worms, dying worms or eggs (with the same antigen stimulating both memory cells and plasma cells for each response). Plasma cells are generated through activation of memory cells by antigen, at a rate governed by the parameter *δ_j_*. Protection is mediated by the plasma cell population, as in the plasma cell-only models. Cross-regulation is shown using dashed lines - it is shown acting in one direction (reducing production of *M*_2_ and *A*_2_, with strength *ρ*_2_) for clarity, but may also act to reduce production of *M*_1_ and *A*_1_. Cross-regulation between the two responses affects both memory and plasma cell production. All parameters are defined in table 1, with parameter values given.

**Table 1:** Parameters used in the models.

Parameter	Meaning	Values used	Units	Source/rationale
Λ*_m_*	Maximum rate of infection	12.5, 25, 50, 100, 200	Worms year^−1^ person^−1^	[39]
*a_c_*	Age above which contact rate stays constant	7.8	Years of age	Fit to data from [39]; see figure 4
*n*	Number of worm compartments in model	1, 9	Compartments	*n* = 1/*n* = 9 for exponential/approximately Gaussian distribution of worm survival respectively
*1/μ*	Natural mean worm life span	3, 6.5, 10	Years	[5], [6]
*κ_j_*	Rate of production of plasma cells in plasma cell model	1	Cells year^−1^ unit antigen^−1^	Variation accounted for in varying immune strength
*σ_j_*	Rate of production of memory B cells in memory model	1	Cells year^−1^ unit antigen^−1^	Variation accounted for in varying immune strength
*δ_j_*	Rate of production of plasma cells from antigen-driven memory cell activation in memory model	1	Plasma cell year^−1^ memory cell^−1^ unit antigen^−1^	Variation accounted for in varying immune strength
*K_m_*	Maximum size of memory population	1000	Cells	Set to limit memory cell growth rate
*γ_j_*	Rate of loss of plasma cells (all models)	0.008, 0.08, 0.8, 8, 80	Cells year^−1^ cell^−1^	[50], [53]
*ω_j_*	Rate of loss of memory B cells	0.008, 0.08, 0.8, 8, 80	Cells year^−1^ cell^−1^	[53], [59]
*θ_j_*	Strength of protection against re-infection	0.00025, 0.001, 0.004, 0.016, 0.064, 0.256, 1.024	Cell^−1^	Broad exploratory range
*ψ_j_*	Strength of immune-mediated worm killing	0.00025, 0.001, 0.004, 0.016, 0.064, 0.256, 1.024	Cell^−1^	Broad exploratory range
*η_j_*	Strength of anti-fecundity response	0.00025, 0.001, 0.004, 0.016, 0.064, 0.256, 1.024	Cell^−1^	Broad exploratory range
*ρ_j_*	Strength of cross-regulation	0.01, 0.1, 1	Unit antigen^−1^	Set to give significant effect on immune development
*β*	Rate of production of ‘cumulative’ response (threshold model)	1	Arbitrary units year^−1^ unit antigen^−1^	Arbitrary constant
*T*	Threshold for cumulative antigen exposure	25, 250	Antigen units	Set to give significant effect on age-intensity curve

Parameter descriptions with all values used, units and sources from the literature where relevant. Antigen units = number of cercariae/live worms/dying worms/eggs as appropriate. ‘Cell’ refers to units of the plasma or memory cell populations. Subscript *j* refers to values for the two different antibody responses (*j* = 1,2).

#### Plasma cell model

The plasma cell-only model (figure 1) describes the development of infection and two antibody responses (modelled as populations of plasma cells) with age in a homogeneous population with endemic schistosome infection. Each antibody response is stimulated by antigen from a single stage of the schistosome life cycle, and each antibody response targets a single life cycle stage in this model. It is assumed that antibody levels are directly proportional to the size of their respective plasma cell populations. This is reasonable, since plasma cells are constitutive producers of antibody, and antibody has a relatively short plasma half-life in humans, of the order of days to weeks [43], [44].

Infection rates are assumed to be the same for all individuals within the population, and to be constant with time, but vary with age as described by equation 1. The age-related contact rate (Λ) is zero at birth, increases linearly with age up to age *a_c_*, when it reaches its maximum level (Λ*_m_*) and remains at this maximum level for all subsequent ages. Worm burden (*P*) is modelled using *n* compartments (equation 2), shown schematically in figure 2. New worms enter the first compartment at a rate Λ(*a*), which can be reduced by anti-reinfection antibody responses as a decreasing exponential function of the number of relevant plasma cells (*A*_1_ or *A*_2_ or both), with relative strength *θ_j_* (*θ_1_* or *θ_2_* as appropriate for the 1^st^ or 2^nd^ (*j* ^th^) antibody response) (equation 2). Worms move between compartments at a constant rate *nμ*, dying naturally when they leave the final (*n* ^th^) compartment, giving a natural death rate *nμP_n_(a)* and an overall mean natural worm life span (in the absence of immunity) of 1/*μ*. Increased immune-induced worm death is included as an additional per-worm death rate, directly proportional to the number of plasma cells and scaled by a factor *ψ_j_* (figure 2 and equation 2). This additional worm death rate applies equally to all worm compartments (meaning that the ability of the immune response to kill adult worms is not affected by worm age). For exponentially distributed worm life span, a single worm compartment is used (*n* = 1), using the equation for *i* = 1 (where *i* is the compartment number) (equation 2). For an approximately Gaussian distribution of worm life spans, nine worm compartments are used (*n* = 9), with the first compartment described by the first line of equation 2, for *i* = 1, and all other compartments described by the second line (for 1<*i*≤*n*). The number of eggs measured in urine (*E*) is assumed to be directly proportional to total worm burden (summed over all worm compartments), and can be reduced by anti-fecundity antibody responses as a decreasing exponential function of the number of plasma cells, with relative strength *η_j_* (equation 3).

The antibody responses are modelled as two separate compartments of plasma cells, *A*_1_ and *A*_2_ (equations 4, 5). Each plasma cell population grows at a rate directly proportional to the level of antigen (*G_j_*) exposure (with the rate governed by parameter *κ_j_*). The antigen stimulating each cell population (*G_j_*) comes from a single stage of the schistosome life cycle (*S_j_*): cercariae, live adult worms, dying adult worms or eggs (equation 6). Each plasma cell population decays at a constant per-cell rate *γ_j_*. Cross-regulation between the two different antibody responses is modelled as a reduction in the production rate of each plasma cell population, proportional to the level of antigen stimulating the other response. This is scaled using a decreasing exponential function, with the strength of cross-regulation governed by the parameter *ρ_j_*.
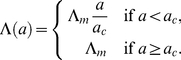
(1)

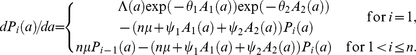
(2)

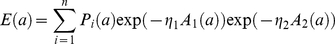
(3)

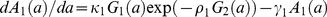
(4)

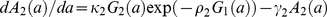
(5)

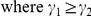


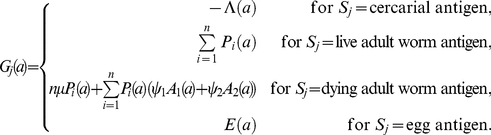
(6)


#### Memory cell model

The memory models include memory B cell populations as well as plasma cells (figure 3). The memory cell populations are assumed to grow through antigen-dependent activation of naive cells, at a rate directly proportional to antigen exposure, with relative strength σ_j_ (equations 7,8). The antigenic source comes from a single life cycle stage (equation 6), and the same stage stimulates the production of both memory cells and plasma cells for each antibody response. A density dependent function with strength K_M_ is used to restrict memory cell population growth. The memory cell populations each decay at a constant per cell rate ω_j_. In the memory models, the plasma cell populations expand through antigen-dependent activation of their respective memory cell populations, governed by the parameter δ_j_, and decay at a constant per-cell rate, γ_j_ (equations 9,10). The effects of the immune response upon the schistosome life stages are mediated by the plasma cells, with no direct effect of memory cells. The infection processes and the impact of antibody upon the parasite life cycle stages are identical to those in the plasma cell-only model (equations 1,2,3,6). Cross-regulation affects the rate of production of both memory and plasma cell populations in these models (with relative strength ρ_j_).
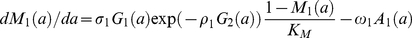
(7)

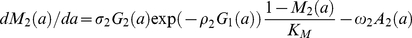
(8)

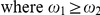


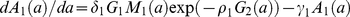
(9)

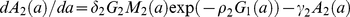
(10)


#### Antigen threshold

For models which include an antigen threshold, a separate equation records cumulative exposure (C) to one of the antigens (G_2_). Cumulative antigen exposure builds up at a rate directly proportional to the level of antigen (with relative strength *β*), and does not decay over time (equation 11). It is assumed that this cumulative antigenic exposure has to exceed a certain threshold level for one of the two immune responses to be stimulated. Preliminary analyses showed that an antigenic threshold can delay the development of an antibody response. Antibody responses which show an age-related ‘switch’ only have a delay in one antibody response, not both, and so an antigen threshold is only applied to one of the responses in this analysis. As different decay rates are used for the two responses (see ‘Model Analysis’ section) the threshold is applied to the response with less rapid decay (A_2_ in this analysis), which is likely to develop later than the more rapidly decaying response [45]. For the plasma cell-only model, A_2_ is not produced if the level of cumulative exposure is below the set threshold. Once this threshold has been exceeded, the plasma cell population grows at a rate proportional to current antigen level (equation 12). At all times, these plasma cells decay at a constant per cell rate γ_2_. The other antibody response, A_1_, is not affected by the antigen threshold, and its production and decay is the same as in the earlier model (equation 4).
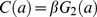
(11)

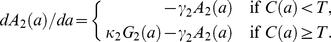
(12)where *T* is a constant.

For the memory model, cumulative exposure is calculated in the same way (equation 11), but the threshold now applies to the more slowly decaying memory response (*M*_2_ in this analysis). *M*_2_ is only made when the level of cumulative exposure to the relevant antigen exceeds the threshold, and is then made at a rate proportional to current antigen levels (equation 13), at all times decaying at a rate *ω*_2_. The equations describing the dynamics of the other memory response and the two antibody responses are the same as previously (equations 8, 9, 10).
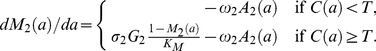
(13)where *T* is a constant.

#### Model analysis

**Figure 4:**
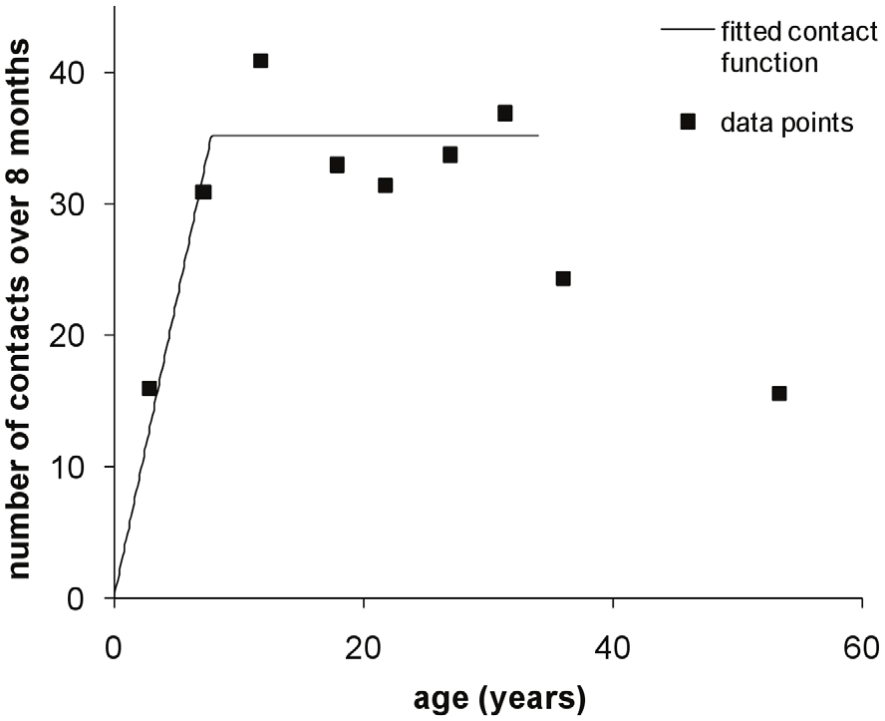
Age-related water contact frequency. Data is for the Burma Valley region in Zimbabwe, with data points extracted from figure 1 in Chan et al. 2000 [39]. The two-part linear function was fitted using least squares.

For both plasma cell-only and memory models, every possible pair wise combination of antigen stimulus (cercariae, live worms, dying worms or eggs) and antibody target (reduced re-infection, increased worm death or reduced fecundity) was used in turn for each of the two antibody responses in a grid-search of the parameter space. All models were run using two different worm survival curves, with worm life spans following exponential or approximately Gaussian distributions (using *n* = 1 or *n* = 9 respectively). Cross-regulation between the two antibody responses was included, operating in one or two directions. An antigen threshold was included in separate models (without cross-regulation). The average worm life span (*L*), taking into account both natural and immune-mediated death, was calculated using equation 14.
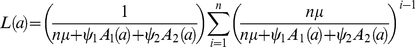
(14)Models were run for values of age from 0 to 34 years old (in line with field data showing stable contact rates in adults up to the age of 34; figure 4). The parameters determining infection rate, antibody strength, antibody and memory decay rates, average worm life span, strength of cross-regulation and threshold level were each varied across plausible ranges, mostly in geometric series (values used are given in table 1), and all possible combinations of these parameters were used in turn. For the plasma cell models, the combinations of plasma cell decay rates used were restricted to those in which *A*_1_ decayed at the same or a faster rate than *A*_2_ (*γ*_1_≥*γ*_2_). For the memory models, it was assumed that both plasma cell populations decayed very rapidly (*γ*_1_ = *γ*_2_ = 80 year^−1^), with different combinations of memory cell decay rates, again restricted to combinations where *M*_1_ decayed at the same or a faster rate than *M*_2_ (*ω*_1_≥*ω*_2_). For each model, parameter combinations were identified which allowed the model to simultaneously meet all of the criteria identified from field data (criteria are detailed in table 2) over a two-fold change in the infection rate (Λ*_m_*).

**Table 2:** Criteria used to judge whether model outputs replicate patterns seen in field data.

Pattern seen in field data	Model criterion	Justification
Peaked age intensity curve	Maximum level of infection occurs between age 6–20 years old	[6], [7], [8], [12], [38], [60], [61]
Reduction of infection level in adults	Infection level at age 34<40% of peak level	[7], [12], [38], [60]
Peak shift	Peak infection intensity is lower and occurs at a later age when infection rate is halved (except for lowest value of Λ*_m_*, where infection intensity is higher and occurs at an earlier age when infection rate is doubled)	[10]
Worm life span	Mean worm life span >1 year in 34 year olds	[5], [6]
Antibody switch	The two antibody responses never simultaneously exceed 30% of their respective maximum levels	[12], [13], [16]
Antibody switch after age of infection peak	Initial antibody response falls below 30% of its maximum level after the age at which maximum infection level recorded	[12], [13]

All criteria must be met over a two-fold change in maximum infection rate (Λ*_m_*) for any parameter set to be deemed successful.

The differential equations were solved numerically using a variable time step fifth-order embedded Runge-Kutta algorithm, adapted from the rkqs routine in Press et al. 2002 [46]. This routine allows both a fourth-order and a fifth-order approximation to be made at each time step, and the difference between the two estimates is used to estimate the truncation error of the fourth-order solution, which is used to determine the size of the next time step taken. Cash-Karp parameters were used for the constants, recommended for their efficiency and error properties [46]. The numerical integration algorithm was implemented in C++.

## Results

### Model criteria

**Figure 5:**
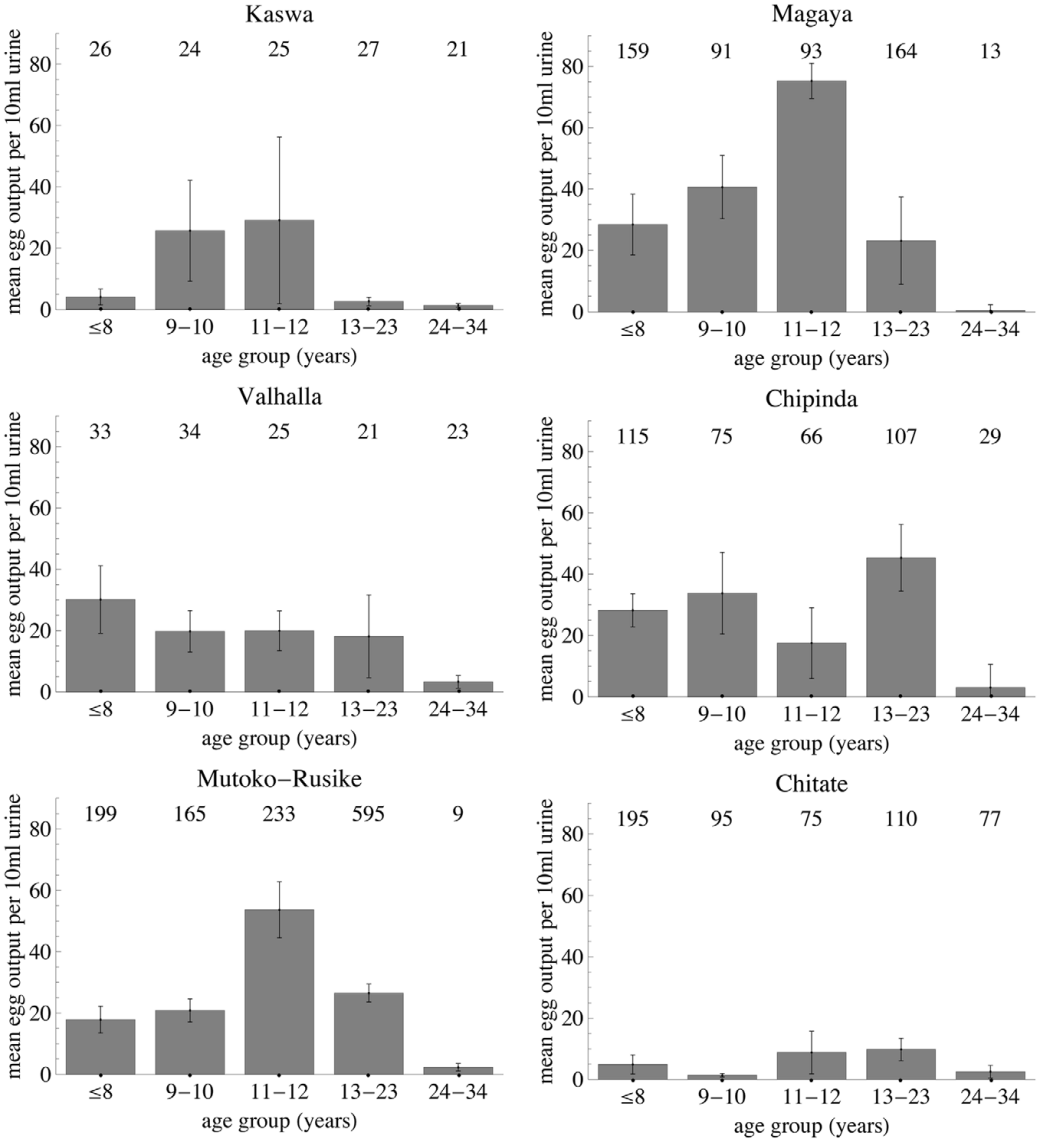
Age-intensity graphs for six Zimbabwean populations with endemic *S. haematobium*. Grey bars represent arithmetic mean egg counts per 10 ml urine for the five age-groups shown, error bars represent standard error of the mean. The numbers at the top of each graph show the sample size for that age group.

Age infection profiles for six Zimbabwean communities with endemic *S. haematobium* infection are shown in figure 5. The key parameters of interest (age and level of peak infection, and ratio between adult and peak infection levels) for these populations are given in table 3. Note that data was only included for individuals up to the age of 34, because data on water contact for the Burma Valley populations showed that this remained fairly stable for adults up to this age, although it declined at older ages (figure 4; [39]). Table 3 also includes information on the infection peak and its relationship to infection in adults for a number of studies of endemic schistosomiasis taken from the literature, which give mean infection intensities by age group, and cover a sufficiently wide age range to capture both the peak and adult levels. The age group in which peak infection occurred ranged between 5–8 for Valhalla and 16–20 [9] or 13–23 for both Chipinda and Chitate. Since 5–8 years was the youngest age group used in the Valhalla analysis, different age groupings were also used to show that infection peaks in the upper end of this age group (data not shown), so that this age range truly reflects where the peak occurs. The median values for these minimum/maximum age groups were used to give an overall estimated range for the age of the peak of 6–18 years old (cf. 12–25 years old for *S. mansoni* [21]). The criterion used for the models uses a range of 6–20 years old, to allow for the fact that wide age ranges were used when grouping adults in these studies (owing to smaller sample sizes), reducing confidence in the actual maximum age of the peak. Adult age groups for comparison with the peak were chosen to be as close to the range used in the analysis in figure 5 as possible. Results from studies giving means of log-transformed egg counts were back-transformed to give geometric means. It is difficult to compare these studies or use all of the estimates for the ratio between adult and peak levels of infection since the different studies use a range of types of means (arithmetic or geometric mean, including the whole population or only those with infection), which give very different answers. From the studies which use arithmetic means for *S. haematobium* infections, the ratio of final∶peak infection level is 0.5–26%. Using different age groupings for the populations shown in figure 5 changed the ratio of final∶peak infection level substantially for some of the populations (Valhalla - ratio up to 34%, Chipinda - ratio up to 30%), so the range used in the model criterion was 0–40% to allow for this instability at the top end of the range.

**Table 3:** Summary of age-intensity profiles for *S. haematobium*.

Reference	Country	Mean	Peak age	Peak level	Adult age	Adult level	% Adult/Peak
Kaswa [12]	Zimbabwe	arithmetic	11–12	29.1	24–34	1.4	4.9
Valhalla [12]	Zimbabwe	arithmetic	5–8	30.2	24–34	3.3	10.9
Mutoko-Rusike [37]	Zimbabwe	arithmetic	11–12	53.7	24–34	2.3	4.3
Magaya [38]	Zimbabwe	arithmetic	11–12	75.3	24–34	0.4	0.5
Chipinda[38]	Zimbabwe	arithmetic	13–23	45.4	24–34	3.0	6.6
Chitate[38]	Zimbabwe	arithmetic	13–23	9.8	24–34	2.5	26.0
[60]	Tanzania	arithmetic	9	311.0	33–34	35.0	11.3
[7]	Zimbabwe	arithmetic	10–12	3405.6	21–40	428.0	12.6
[7]	Zimbabwe	arithmetic	7–9	853.4	21–40	20.7	2.4
[7]	Zimbabwe	arithmetic	10–12	922.3	21–40	55.9	6.1
[7]	Zimbabwe	arithmetic	7–9	688.3	21–40	18.2	2.6
[7]	Zimbabwe	arithmetic	10–12	373.7	21–40	43.0	11.5
[62]	Kenya	geometric (all)	11–15	25.2	31–40	3.8	15.0
[61]	Nigeria	geometric (all)	10–14	11.6	30–39	7.4	64.2
[6]	Gambia	geometric (all)	8–12	326.0	25–39	1.9	0.6
[6]	Gambia	geometric (all)	8–12	170.0	25–39	1.8	1.1
[8]	Gambia	geometric (all)	5–9	108.6	15+	11.0	10.1
[9]	Zimbabwe	geometric (positives)	16–20	234.7	31–50	32.4	13.8
[9]	Zimbabwe	geometric (positives)	7–9	116.3	31–40	11.1	9.5
[13]	Zimbabwe	geometric (positives)	10–14	31.0	25–44	4.3	14.0
[63]	Kenya	geometric (unspecified)	12–15	34.9	adults	6.0	17.1

**Figure 6:**
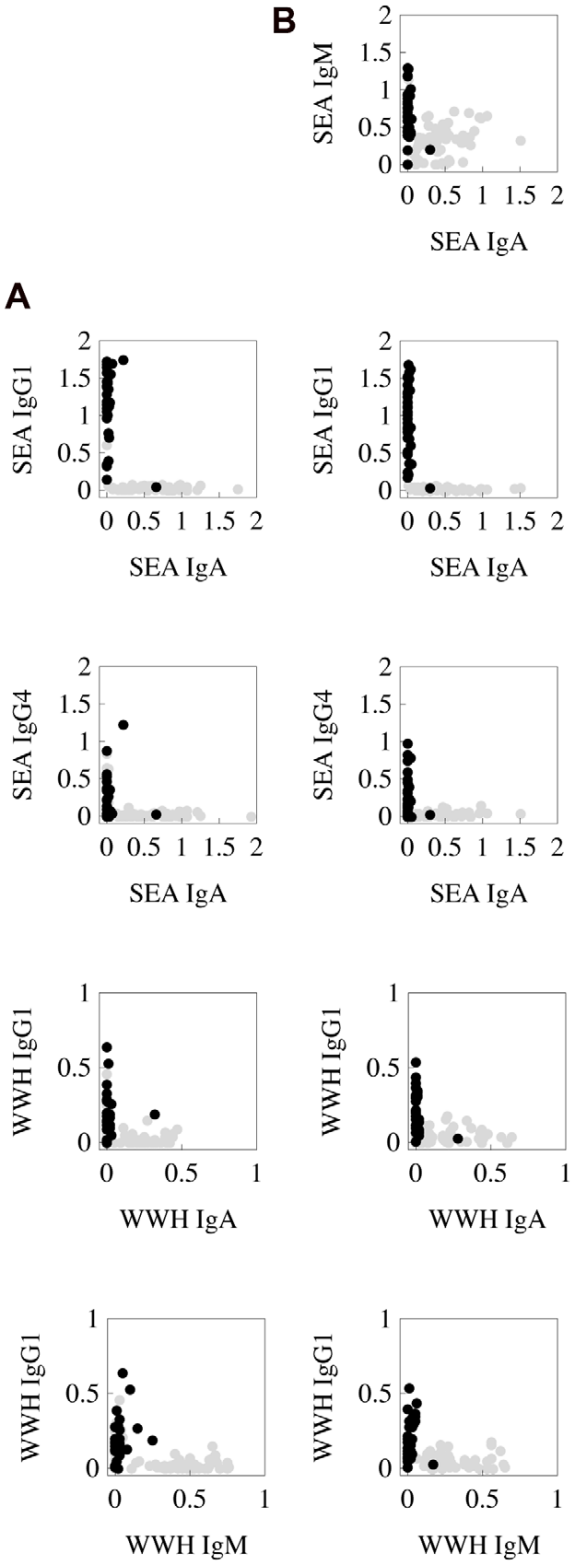
Changes in co-distributions between antibodies for which a switch is seen, by age group, for (A) Kaswa and (B) Valhalla. Grey circles: 0–14 year olds, black circles: 15–34 year olds.

Testing for significant negative correlations between different antibody isotypes across the full population age range (using an age range of 0–34 as in the previous analyses) was able to discriminate well the dichotomous antibody relationship indicative of an antibody switch. In the Burma Valley populations, these negative correlations all occurred between isotypes which changed significantly with age in opposite directions - i.e. one of the pair increased with age and the other decreased (as assessed by Kruskal Wallis test, using age as a categorical variable in the five age groups used for the infection profiles). In Kaswa, significant negative correlations were found between the IgG4 and IgA anti-SEA responses, IgG1 and IgA anti-WWH responses and the IgM and IgG1 anti-WWH responses as well as the SEA IgA and IgG1 antibodies (figure 6A). In Valhalla, all of these correlations were also seen, and additionally a negative correlation between IgA and IgM SEA antibodies (figure 6B). For the negative correlations which were significant at the 1% level, the correlation coefficient varied between −0.662 and −0.247. In Mutoko-Rusike, there were no significant negative correlations between different isotypes directed at the same antigen preparation (for cercarial, egg or adult worm antigen preparations). Most antibodies were significantly positively correlated with each other (data not shown). This may have been because relatively few adults were included in this dataset. In a further published study [13], for which the raw data were not available, negative correlations were also reported between antibody responses which have opposite trends with age: SEA IgE and SEA IgG4, SEA IgE and SEA IgM, and WWH IgA and WWH IgM [13], strongly suggesting similar antibody switch patterns.

Because testing for a correlation was not suitable for detecting an antibody switch in the deterministic models used here, an alternative metric was used to construct the antibody switch criterion: that the two antibody responses never simultaneously exceed n% of their respective maximum levels. A suitable level for n was determined by selecting 10 parameter sets which passed the criterion only at each of the following cut-offs: 40%, 30%, 20%, 10%, 5%, and running a stochastic individual-based model based upon the same model framework to check the correlation across different ages. While those which passed at 5% or 10% always gave a highly significant negative correlation in the stochastic version of the model, those which passed at 20% or 30% occasionally did so as well, and so a conservative threshold of 30% was chosen. For all of the antibody switches detected in the datasets, the antibody switch occurred after the age of infection intensity and so this was used as an additional criterion. The final set of model criteria is given in table 2.

### Model results

The combination of both the parasite life cycle stage providing the main antigenic stimulus for each antibody response, and the stage being targeted by the antibody responses (‘antigen:target combination’), was critical in determining whether or not the models were able to reproduce all of the patterns identified from field data. For this reason, results are presented and analysed by antigen:target combination within each model structure. For each model structure (described in the subsections below), the main outputs of interest were the number and consistent features of antigen:target combinations for which all of the model criteria could ever be met, and the proportion of total parameter space explored for which all of the criteria were met. Note that the number of antigen:target combinations meeting the criteria for each model structure should not be taken as an indication of how ‘good’ each model is. The proportion of parameter space explored for which all criteria were met gives some indication of how well each model agrees the data (although this should be interpreted with caution – see Discussion section). Proportions rather than absolute numbers were used because different numbers of parameter sets were explored for each model.

#### Plasma cell models without cross-regulation or thresholds

**Figure 7:**
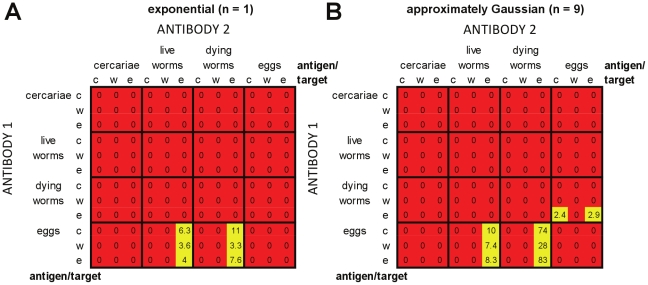
Relative success of plasma cell models without cross-regulation or thresholds in reproducing infection and antibody profiles seen in field data. Individual entries give the number of parameter sets per 1000 tested for which all of the criteria (as laid out in table 2) were met, for each different antigen:target combination for the two antibody responses. The total number of parameter combinations tested for each antigen:target combination was 11,025. Antigens and targets for *A*_2_ (which has equal or slower decay than *A*_1_) are given along the *x*-axis of the table, antigens and targets for *A*_1_ are given along the *y*-axis. Targets: c = cercariae (reduced re-infection); w = worms (increased worm death); e = eggs (reduced fecundity). Red squares indicate that no parameter combinations were ever found for which all criteria were met; orange that fewer than 1 in 1000 parameter combinations were found that could meet all criteria, and yellow that more than 1 in 1000 parameter combinations were able to meet all criteria. No cross-regulation or thresholds were included in these models. In panel (A) models used exponentially-distributed worm survival (*n* = 1). In panel (B) models used approximately Gaussian-distributed worm survival (*n* = 9).

Figure 7 shows results for the plasma cell-only models without cross-regulation or antigen thresholds. For each of the possible antigen:target combinations of the two antibody responses, the proportion of parameter sets tested over which models were able to simultaneously reproduce all of the required patterns is shown. Results are shown separately for models with exponentially distributed worm life span (*n* = 1; figure 7A), and with approximately Gaussian-distributed worm life span (*n* = 9; figure 7B). In the absence of either cross-regulation or an antigen threshold, only a small number of antigen:target combinations were able to reproduce all of the patterns for any part of the parameter space tested, 6/144 for *n* = 1 and 8/144 for *n* = 9. These models all had one antibody response stimulated by antigens from live or dying worms, which reduced worm fecundity, with the other antibody response stimulated by egg antigens. In all cases, the antibody response stimulated by eggs peaked early and was replaced by the other, worm-stimulated, response. Usually the egg-stimulated response decayed more rapidly than the worm-stimulated response (models with egg-stimulated *A*_1_ and worm-stimulated *A*_2_), but for some of the models with Gaussian-distributed worm life span and dying worm antigen, this was reversed (models with worm-stimulated *A*_1_ and egg-stimulated *A*_2_). For the antigen:target combinations which were ever able to reproduce the required field patterns, a very restricted part of parameter space (≤8.3% of combinations tested) gave results which passed all of the criteria. Using approximately Gaussian distributed worm survival (figure 7B) increased the range of parameters for which it was possible to pass all of the criteria when compared with exponentially distributed worm survival (figure 7A). Across the whole parameter space tested, only 0.25 parameter sets per 1000 tested were able to meet all of the criteria for models with exponentially distributed worm survival, compared with 1.5 per 1000 tested for approximately Gaussian distributed worm survival.

#### Memory models without cross-regulation or thresholds

**Figure 8:**
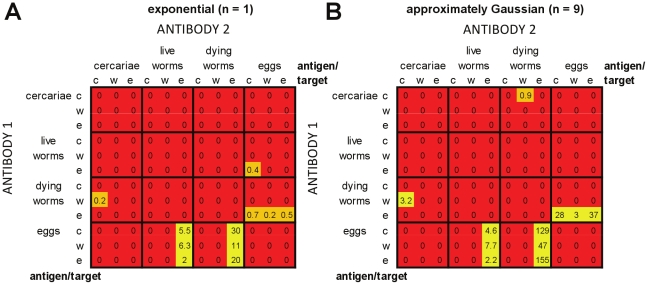
Relative success of memory models without cross-regulation or thresholds in reproducing infection and antibody-profiles seen in field data. See legend for figure 7. In this model, it is the memory cell populations for each antibody response which differ in their decay rates – *M*_2_ has equal or slower decay than *M*_1_.

Figure 8 shows the same results for memory models without cross-regulation or an antigen threshold. As in the plasma cell-only models, a small number of antigen:target combinations were able to reproduce all of the field patterns (11/144 for both *n* = 1 and *n* = 9). Most of these, like the plasma cell-only models, had one fecundity-reducing worm-induced antibody response while the other response was stimulated by egg antigen. A small number of models had a different combination of antigens and targets, with one response stimulated by antigens from dying worms, which reduced worm survival, and the other stimulated by cercarial antigens and reducing reinfection.

For the antigen:target combinations which were ever able to reproduce the required field patterns, only a limited range of the parameter space (≤15% of combinations tested) gave results which passed all of the criteria. As with the plasma cell models, using approximately Gaussian distributed worm survival (figure 8B) increased the range of parameters for which it was possible to pass all of the criteria when compared with exponentially distributed worm survival (figure 8A). Across the whole parameter space tested, only 0.54 parameter sets per 1000 tested were able to meet all of the criteria for models with exponentially distributed worm survival, compared with 2.9 per 1000 tested for approximately Gaussian distributed worm survival.

#### Plasma cell models with cross-regulation

**Figure 9:**
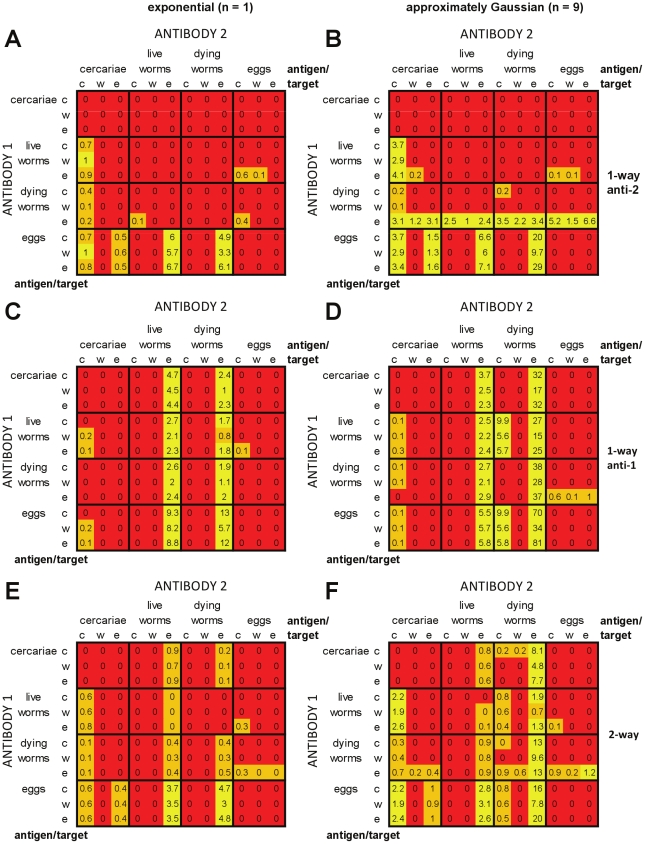
Relative success of plasma cell models with cross-regulation in reproducing infection and antibody profiles seen in field data. See legend for figure 7. For panels (A) and (B), there is one-way down-regulation of the less rapidly decaying antibody response (*A*_2_) in panels (C) and (D) there is one-way down-regulation of the more rapidly decaying antibody response (*A*_1_) and in panels (E) and (F) there is two-way cross-regulation of both antibody responses. The total number of parameter combinations tested for each antigen:target combination was 33,075 for models with one-way regulation (A–D) and 99,225 for models with two-way cross-regulation (E,F). The left-hand set of panels are for models with exponentially-distributed worm life span (*n* = 1) (A,C,E), the right-hand set are for models with approximately Gaussian-distributed worm life span (*n* = 9) (B,D,F).

Figure 9 shows the impact of cross-regulation in plasma cell-only models, acting in one or both directions. Inclusion of cross-regulation increased the number of antigen:target combinations which could meet the criteria. Across all of the cross-regulation models (figure 9), 57/144 (40%) of the different antigen:target combinations tested were ever able to reproduce the infection and antibody patterns. For the models that were able to reproduce these field patterns, one of the antibody responses always fell into one of two broad groupings of antigen:target combinations: (i) antigen cercariae/live worms/dying worms, target fecundity; or (ii) antigen cercariae/dying worms, target re-infection. Models with cross-regulation of both responses enabled the greatest number of different antigen and target combinations to reproduce field patterns at least once (figure 9E,F). Models with approximately Gaussian-distributed worm survival were able to replicate field patterns for a greater number of antigen:target combinations than equivalent models with exponential worm survival.

Models with cross-regulation of the more rapidly decaying response (*A*_1_) were able to reproduce patterns seen in the field over the greatest proportion of parameter space (figure 9C,D), with 0.69 parameter sets per 1000 tested able to meet all of the criteria for models with exponentially distributed worm survival, and 3.6 per 1000 tested for approximately Gaussian distributed worm survival. Similarly, for both models with cross-regulation of the more slowly decaying response (*A*_2_) and models with cross-regulation of both responses, using approximately Gaussian distributed worm survival increased the range of parameters for which it was possible to pass all of the criteria when compared with exponentially distributed worm survival.

#### Memory models with cross-regulation

**Figure 10:**
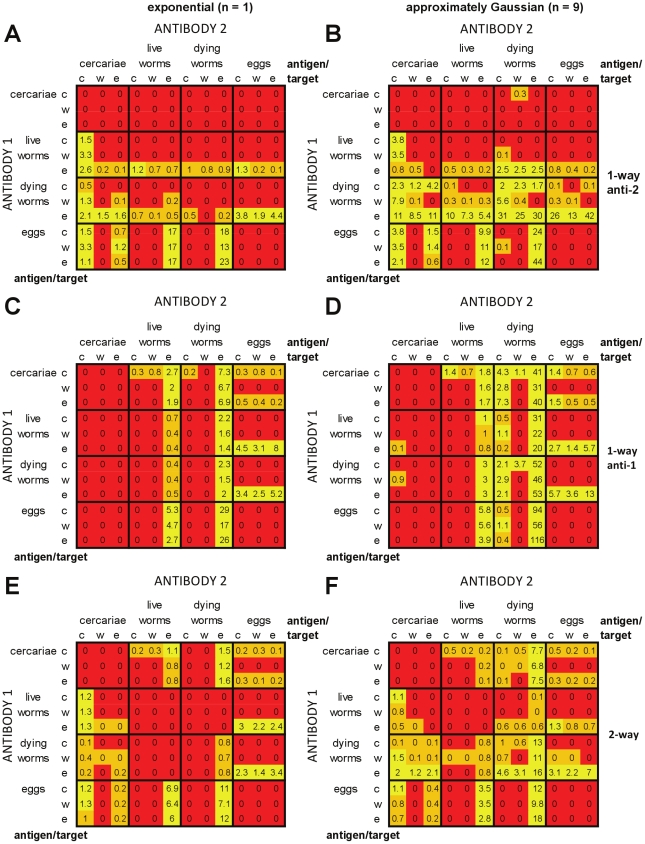
Relative success of memory models with cross-regulation in reproducing infection and antibody profiles seen in field data. See legends for figures 4 and 6. In this model, it is the memory cell populations for each antibody response which differ in their decay rates – *M*_2_ has equal or slower decay than *M*_1_.

Similar overall patterns were seen for memory models with cross-regulation (figure 10). Inclusion of cross-regulation increased the number of antigen:target combinations which could meet the criteria. Across all of the cross-regulation models tested, 87/144 (60%) of the different antigen:target combinations tested were ever able to reproduce all of the field patterns. For the models that were able to reproduce these field patterns, one of the antibody responses always fell into one of the three following broad groupings: (i) antigen cercariae/live worms/dying worms, target fecundity; (ii) antigen cercariae/dying worms, target re-infection; or (iii) antigen dying worms; target worm survival (unique to the memory models). Models with cross-regulation of both responses enabled the greatest number of different antigen and target combinations to reproduce field patterns at least once (figure 10E,F). Models with approximately Gaussian-distributed worm survival were again able to replicate field patterns for a greater number of antigen:target combinations than equivalent models with exponential worm survival.

Models with cross-regulation of the more rapidly decaying response (*M*_1_) were able to reproduce patterns seen in the field over the greatest proportion of parameter space (figure 10C,D), with 1.1 parameter sets per 1000 tested able to meet all of the criteria for models with exponentially distributed worm survival, and 4.9 per 1000 tested for approximately Gaussian distributed worm survival. Similarly, for both models with cross-regulation of the more slowly decaying response (*M*_2_) and models with cross-regulation of both responses, using approximately Gaussian distributed worm survival increased the range of parameters for which it was possible to pass all of the criteria when compared with exponentially distributed worm survival.

Memory models were able to reproduce field patterns for a greater number of possible antigen:target combinations and over a greater overall portion of parameter space than were equivalent plasma cell models (figure 10 cf. figure 9).

#### Plasma cell models with an antigen threshold

**Figure 11:**
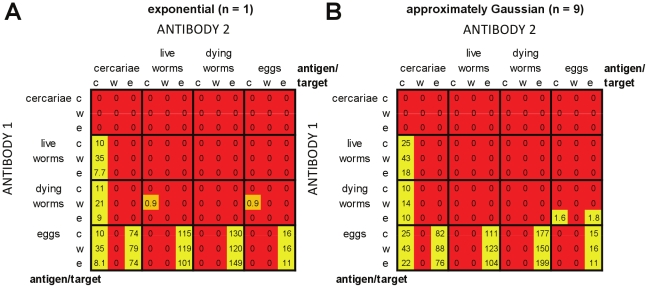
Relative success of plasma cell models with an antigen threshold in reproducing infection and antibody profiles seen in field data. See legend for figure 7. A threshold is included for the less rapidly decaying antibody response (*A*_2_). The total number of parameter combinations tested for each antigen:target combination was 22,050.

The inclusion of an antigen threshold also influenced whether models could reproduce field patterns. For plasma cell models, inclusion of an antigen threshold on the more gradually decaying response (*A*_2_) increased the number of antigen:target combinations which could meet the criteria (figure 11). The number of antigen:target combinations which were able to reproduce all of the patterns for any part of the parameter space tested was increased to 23/144 (17%) for both *n* = 1 and *n* = 9. For the models that were able to reproduce these field patterns, one of the antibody responses always fell into one of the two following broad groupings: (i) antigen cercariae/live worms/dying worms/eggs, target fecundity; or (ii) antigen cercariae, target re-infection. Note that the anti-fecundity response stimulated by egg antigens is unique to the threshold models.

For the antigen:target combinations which were ever able to reproduce the required field patterns, up to 20% of the parameter combinations tested now gave results which passed all of the criteria. Using approximately Gaussian distributed worm survival (figure 11B) slightly increased the range of parameters for which it was possible to pass all of the criteria when compared with exponentially distributed worm survival (figure 11A). Across the whole parameter space tested, 8 parameter sets per 1000 tested were able to meet all of the criteria for models with exponentially distributed worm survival, compared with 9.5 per 1000 tested for approximately Gaussian distributed worm survival.

#### Memory cell models with an antigen threshold

**Figure 12:**
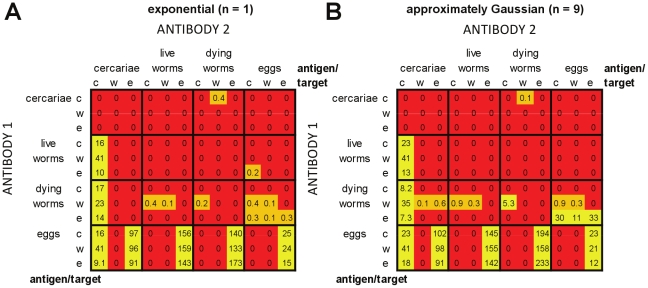
Relative success of memory models with an antigen threshold in reproducing infection and antibody profiles seen in field data. See legend for figure 11. In this model, it is the memory cell populations for each antibody response which differ in their decay rates – *M*_2_ has equal or slower decay than *M*_1_.

Similar patterns were seen for memory models with an antigen threshold on the more gradually decaying response (*M*_2_) (figure 12). The number of antigen:target combinations which were able to reproduce all of the patterns for any part of the parameter space tested was increased to 31/144 (22%) for *n* = 1 and 32/144 (22%) for *n* = 9. For the models that were able to reproduce these field patterns, one of the antibody responses always fell into one of the three following broad groupings: (i) antigen cercariae/live worms/dying worms/eggs, target fecundity; (ii) antigen cercariae, target re-infection; or (iii) antigen dying worms, target worm survival (unique to the memory models). The anti-fecundity response stimulated by egg antigens is unique to the threshold models.

For the antigen:target combinations which were ever able to reproduce the required field patterns, up to 23% of parameter combinations tested now gave results which passed all of the criteria. Using approximately Gaussian distributed worm survival (figure 12B) slightly increased the range of parameters for which it was possible to pass all of the criteria when compared with exponentially distributed worm survival (figure 12A). Across the whole parameter space tested, 10 parameter sets per 1000 tested were able to meet all of the criteria for models with exponentially distributed worm survival, compared with 11.6 per 1000 tested for approximately Gaussian distributed worm survival.

For models including an antigen threshold, memory models (figure 12) were able to reproduce field patterns for a greater number of possible antigen:target combinations and over a greater overall portion of parameter space than were equivalent plasma cell models (figure 11).

#### Models which never meet all criteria

Analysing the results by antigen:target combination, it was found that some of these were never able to reproduce all of the field patterns in any of the different models tested. Altogether, 54 of the 144 different antigen:target combinations tested failed to ever pass all of the criteria simultaneously. These combinations are listed in table 4. These models are ordered by the *A*_2_ response, which tended to be the stronger determinant of model success. Within this set of antigen:target combinations, dying worms were less likely to be the antigenic stimulus for either response than other stages of the life cycle, and for more than half of them, the target of the longer-lived *A*_2_ response was reduced worm survival.

**Table 4:** Combinations of life cycle stage providing the main source of antigens for, and life cycle stage targeted by, each antibody response, for which the model criteria were never met for any parameter combination.

Life cycle stage			
Antigen for *A*_2_	Targeted by *A*_2_	Antigen for *A*_1_	Targeted by *A*_1_
cercariae	cercariae	cercariae	cercariae
cercariae	cercariae	cercariae	worm survival
cercariae	cercariae	cercariae	eggs
cercariae	worm survival	cercariae	cercariae
cercariae	worm survival	cercariae	worm survival
cercariae	worm survival	cercariae	eggs
cercariae	worm survival	live worms	cercariae
cercariae	worm survival	live worms	worm survival
cercariae	worm survival	eggs	cercariae
cercariae	worm survival	eggs	worm survival
cercariae	worm survival	eggs	eggs
cercariae	eggs	cercariae	cercariae
cercariae	eggs	cercariae	worm survival
cercariae	eggs	cercariae	eggs
cercariae	eggs	live worms	cercariae
cercariae	eggs	live worms	worm survival
live worms	cercariae	cercariae	worm survival
live worms	cercariae	cercariae	eggs
live worms	cercariae	live worms	cercariae
live worms	cercariae	live worms	worm survival
live worms	cercariae	eggs	cercariae
live worms	cercariae	eggs	worm survival
live worms	cercariae	eggs	eggs
live worms	worm survival	cercariae	worm survival
live worms	worm survival	cercariae	eggs
live worms	worm survival	live worms	cercariae
live worms	worm survival	live worms	worm survival
live worms	worm survival	dying worms	cercariae
live worms	worm survival	eggs	cercariae
live worms	worm survival	eggs	worm survival
live worms	worm survival	eggs	eggs
dying worms	worm survival	cercariae	eggs
dying worms	worm survival	live worms	cercariae
dying worms	worm survival	live worms	worm survival
dying worms	worm survival	eggs	cercariae
dying worms	worm survival	eggs	worm survival
dying worms	worm survival	eggs	eggs
eggs	cercariae	cercariae	worm survival
eggs	cercariae	live worms	cercariae
eggs	cercariae	live worms	worm survival
eggs	cercariae	eggs	cercariae
eggs	cercariae	eggs	worm survival
eggs	cercariae	eggs	eggs
eggs	worm survival	cercariae	worm survival
eggs	worm survival	live worms	cercariae
eggs	worm survival	live worms	worm survival
eggs	worm survival	dying worms	cercariae
eggs	worm survival	eggs	cercariae
eggs	worm survival	eggs	worm survival
eggs	worm survival	eggs	eggs
eggs	eggs	cercariae	worm survival
eggs	eggs	live worms	cercariae
eggs	eggs	live worms	worm survival
eggs	eggs	dying worms	worm survival

#### Immune cell decay rates in successful models

Due to the balanced study design, which used every potential parameter combination, it was possible to identify preferred parameter values from their relative frequencies in models which met all of the criteria. Results are presented for the decay rates for the immune cells (plasma cells and memory B cells), which are estimable (at least in principle). The relative rates of decay for the two antibody responses are of interest as well as absolute values.

**Figure 13:**
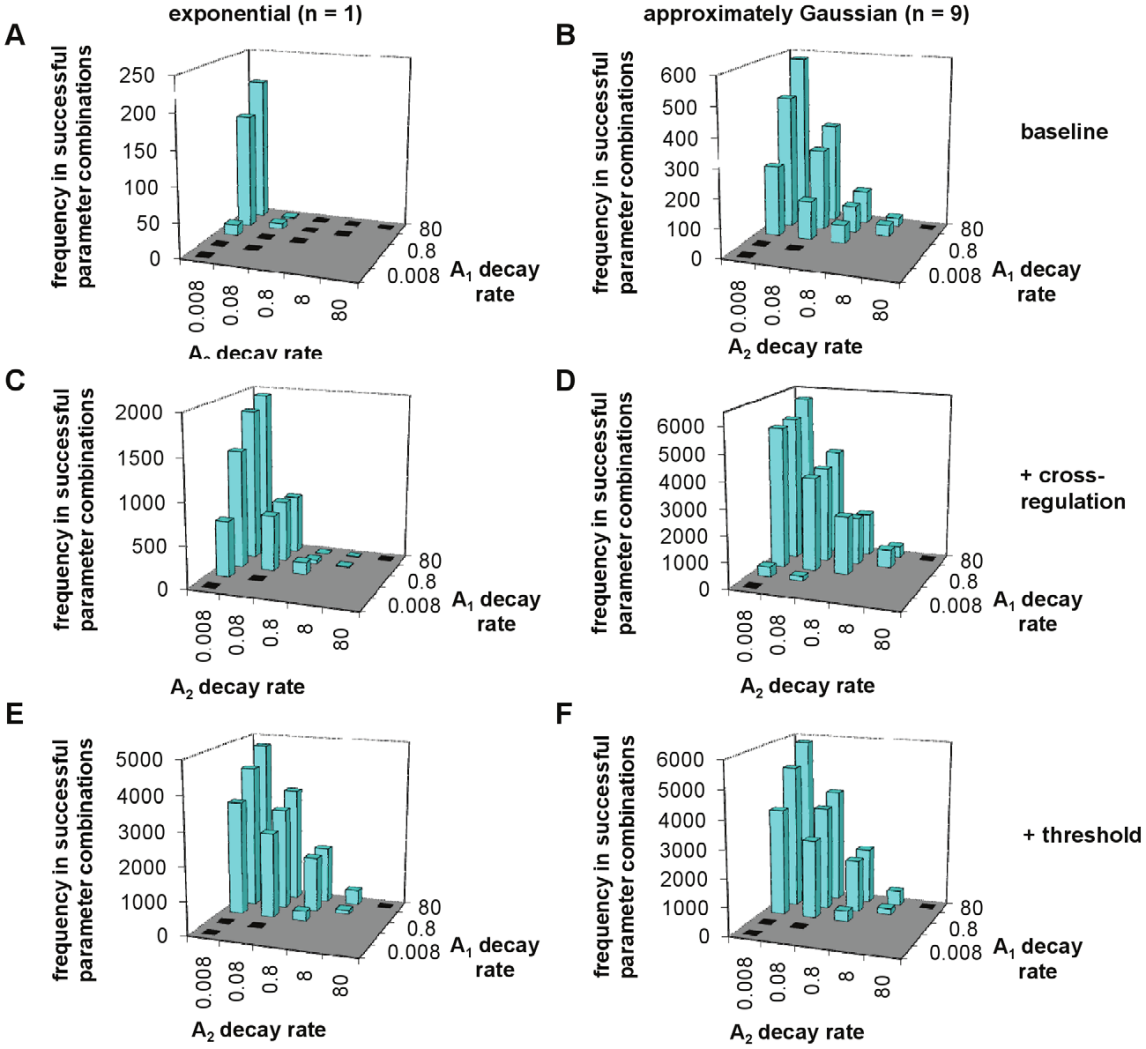
Antibody decay rates for plasma cell models which pass all criteria. Plots show the total number of times parameter combinations including the different possible combinations of decay rates for the two plasma cell populations pass all criteria. (A,B) Models without cross-regulation or thresholds, (C,D) cross-regulation models (total frequencies summed over all of them), (E,F) models with a threshold on *A*_2_. The left-hand panels are for models with exponentially-distributed worm life span (*n* = 1) (A,C,E), the right-hand ones are for models with approximately Gaussian-distributed worm life span (*n* = 9) (B,D,F). All of the different combinations of decay rates that were used have a bar on the chart; black bars indicate that no successful parameter combination had this combination of antibody decay rates, blue bars that at least one successful parameter combination had this combination of antibody decay rates. Note that different maximum values are used on the *z* (frequency)-axis.

For plasma cell-only models with exponential worm life span and without cross-regulation or an antigen threshold, distinct combinations of antibody survival were found to work (figure 13A). For these models to meet all of criteria, it was necessary to have one antibody response with very slow decay (*A*_2_ decay of 0.008 or 0.08 year^−1^, equivalent to a half-life of 9–90 years) with very rapid decay of the other response (*A*_1_ decay of at least 0.8 year^−1^, or a half-life of 10 months or less), with at least a 100-fold difference between decay rates for the two responses. Inclusion of one or more of Gaussian-distributed worm survival (figure 13B,D,F), cross-regulation (figure 13C,D) or an antigen threshold (figure 13E,F) in the plasma cell-only models increased the range of survival rates which were seen for the ‘longer-lived’ *A*_2_ response, and allowed field patterns to be reproduced when the two antibody responses had equal decay rates. In all of these models there was still an overall preference for having a disparity between the decay rates of the two plasma cell populations.

For the memory models, plasma cell decay rates were kept constant at 80 year^−1^ (half-life of 3 days) for both responses, and decay rates for memory cell populations were varied. Less clear patterns were seen in memory decay rates (figure S1). There was a consistent preference for slower decay rates of *M*_2_, but preferred values for *M*_1_ varied widely by model. For all of the models, it was possible to reproduce all of the field patterns when the two memory responses had equal decay rates.

#### Natural mean worm life span in successful models

The distribution of natural mean worm life span (not taking into account anti-worm immunity) was also investigated in successful models, to see what influence this had upon the ability of the models to reproduce field patterns. These results are shown in figures S2 and S3. The preferred worm life span varied with the antigen stimulating *A*_2_, the worm survival distribution and whether or not an antigen threshold was included. For example, in models with cross-regulation, if live or dying worms stimulated *A*_2_, there was a preference for longer-lived worms, but with cercarial antigen, there was a preference for a short worm life span if worm survival was exponentially distributed. Since all of these models were able to pass all of the criteria, it is not possible to conclude from this that any particular value for worm life span is more consistent with patterns seen in field data.

#### Importance of different criteria

The relative importance of the different criteria used was assessed by looking at how frequently they were passed, singly or in pairs, and at how often they were responsible for excluding parameter combinations. This was done by summing up the number of times each criterion was the only one to be failed by any of the parameter sets tested. Only 0.21% of all of the parameter sets tested passed all six criteria over a two-fold change in maximum infection rate. Numbers passing the different criteria (singly or in pairs) were assessed prior to applying the two-fold infection rate change condition. Overall, the antibody switch after the infection peak was the criterion passed least often (passed by 4% of all parameter sets tested); several antigen:target combinations never reproduced the antibody switch after the peak. Next came the infection level in adults relative to the peak (passed by 28% of parameter sets) and the age of the peak (29%). These figures varied considerably by model structure. The worm lifespan criterion was only failed by models with anti-worm immunity (in either antibody response). The different criteria were not independent of one another. In particular, the antibody switch occurring after the peak was dependent upon an antibody switch occurring at all, meaning high levels of association between these two criteria, although in models with cross-regulation the criterion for the antibody switch occurring after the infection peak excluded a large number of models which were able to reproduce the antibody switch. Additionally, parameter sets passing the criterion relating to reduced infection intensity in adults were consistently more likely to give an antibody switch after the infection peak than would be expected if these criteria were independent. A less marked, but consistently positive, association was also seen between passing the peak age and peak shift criteria. Conversely, a negative association was seen between the following criteria: between peak age and reduction of infection in adults (except in the two-way cross-regulation models), and between the peak age and antibody switch after the peak criteria. This trade-off between the peak age and antibody switch after the peak criteria, which were two of the most-frequently failed criteria, led to only 0.54% of parameter sets passing both of these criteria simultaneously. It was found that most parameter combinations failed on multiple criteria, with only 6.4% of those that failed failing to pass only a single criterion. Each of the six criteria excluded some parameter sets single-handedly, demonstrating that they were all discriminatory in this analysis.

## Discussion

It was shown that testing models for their ability to simultaneously reproduce multiple patterns seen in both infection and antibody data enabled a large number of potential model structures and parameter combinations to be rejected. These results give insight into the likely mechanisms giving rise to these patterns. It was found that both the stage of the life cycle which provided the main antigenic stimulus for each antibody response, and the stage of the life cycle which was targeted by a protective antibody response were critical in determining whether the models could reproduce infection and antibody profiles consistent with field data. Some of the models which had dying worms as the main source of protective antigen were able to reproduce all of the required patterns. However, some models which had other stages of the life cycle acting as the principal antigen source could also meet all of the criteria, suggesting that it is not necessary for dying worms to provide the main source of protective antigen to explain the patterns explored here. Inclusion of cross-regulation, an antigen threshold or using approximately Gaussian-distributed worm survival each increased the number of antibody antigen-target combinations, and the range of parameter space for which it was possible to reproduce patterns seen in the field, but none of these were essential for enabling all of the field patterns to be replicated. Other authors fitting models to data ranges using intensive Latin hypercube sampling of the parameter space suggest that the number of successful parameter sets gives an indication of how good a fit the model is [47]. Since we sampled the parameter space in a less intensive way here, this relationship may not be as robust, but nonetheless suggests that threshold models, models with down-regulation of the longer-lived antibody response, and models with dying worm antigens (in tandem with non-exponential worm survival) are in better agreement with the data than other models. From this analysis it is possible to exclude some combinations of life cycle stage providing the main antigenic stimulus for, and life cycle stage targeted by, each antibody response, and these combinations are listed in table 4. While previous modelling had demonstrated that different combinations of the stage acting as stimulus and target could give rise to qualitatively different population infection profiles [11], the main differences identified there related to relative levels of infection in older age groups across different areas of varying transmission, which there is still insufficient data to test. It was interesting to see that, here, many combinations with immune-induced worm death were excluded, mainly on the basis of a very short resulting worm lifespan, and that many of the models with dying worms as the main source of protective antigen were retained, adding support for this hypothesis.

The approach used here was a type of pattern-oriented modelling, testing a range of different model structures and parameter values for their ability to simultaneously reproduce multiple patterns seen in population data [23]. Previous modelling studies have shown that individual patterns in macroparasite infection data can frequently be reproduced by more than one mechanism or combinations of mechanisms, particularly the peaked age-intensity curve and the peak shift [11], [21], [22]. In this study, ‘successful’ models had to simultaneously pass six different criteria for infection and antibody profiles, including the peaked age-intensity curve (within limits identified from field data), the peak shift, an antibody ‘switch’ and realistic mean worm survival, making it much more likely that these models incorporate the ‘true’ mechanisms determining field patterns. It was shown that all of the criteria used were informative, as they were all able to exclude some parameter combinations single-handedly. Thorough sensitivity analysis, using a balanced design where all possible combinations of levels of different parameters were investigated, increases confidence that all of the model structures which can possibly reproduce these patterns have been identified here.

Although the immune responses are represented in a simplistic fashion in these models, they should capture the important features of the antibody response, being based upon well characterised B cell maturation pathways [48], and covering the main alternative theories for the persistence of antibody: (i) antigen-independent mechanisms either through persistence of long-lived plasma cells or continual non-specific activation of memory cells to form short-lived plasma cells (both represented by the plasma cell-only models), and (ii) antigen-dependent mechanisms, with specific antigenic activation of memory cells to form short-lived plasma cells (memory models) [49], [50].

This approach was intended to identify the essential mechanisms underlying universal patterns of schistosome infection and antibody, by selecting models that give outputs consistent with a wide range of studies of endemic *S. haematobium*, rather than trying to accurately replicate patterns seen in individual studies. To this end, patterns were chosen that were seen across multiple different studies. However, some of these patterns are more robust than others; the age of the infection peak and the peak shift have been well characterised across numerous different studies, but fewer studies have reported specific antibody responses by age. The antibody switch has been clearly identified in three Zimbabwean populations [12], [13], [16], but was not seen in data from a fourth study [36]. This pattern has not been specifically looked for in other published studies, meaning that it may not be universal. This would prevent the generalisability of the findings to populations where an antibody switch is not seen. However, the antibody switch was seen across a number of different isotypes in all three of these populations, making it a robust and striking pattern within these populations which deserves exploration. The use of semi-quantitative criteria drawn from different studies also precluded the use of more statistical or likelihood-based model fitting techniques, which may be used when trying to replicate a particular data series [51], but are not suitable in this instance, where we draw on evidence from numerous distinct data sets.

It was of interest to note that many of the models required a large difference in decay rates between the two immune responses. The maximum estimated half-life of 9–90 years for the late response (*A*_2_ or *M*_2_) is in line with estimates for the half-life of long-lived plasma cells (23 years; [52]) and the half-life of vaccinia-specific antibody responses in the absence of re-exposure (92 years; [53]). The minimum estimated half-life of 5–46 days for the early antibody response (*A*_1_ or *M*_1_) fits with estimates for short-lived plasma cell half-life (3–10 days, [50]).

A potential limitation of these models is that they describe population averages without taking into account distributions of infection or antibody. The assumption that all individuals within a certain population are exposed at the same rate is unlikely to be true, as most studies of water contact (a good proxy for infection rate) show highly over-dispersed contact patterns between individuals over set periods of time [54], [55]. While the assumption of homogeneous contact rates is not likely to affect the conclusions drawn about the factors underlying the antibody switch and other qualitative population level patterns, it may affect the precise quantitative conclusions drawn, and also prevents patterns in the distribution of infection or antibody from being used as additional criteria. A good way to look at the effects of heterogeneous contact rates for these types of models is through the use of fully stochastic individual based models (IBMs) [39], [56], which would also allow patterns of aggregation of infection (and antibody) to be simulated and compared to field data, providing additional criteria against which to test the models. However, it was not feasible in this study to cover the same breadth of model structures and parameter ranges with IBMs, which take longer to run and need to be repeated many times to account for stochastic variation in individual simulations. A further pattern which could also be used to differentiate between different model structures is the observation that praziquantel treatment leads to a sustained antibody ‘switch’ in younger children similar to that seen occurring naturally with age [12].

The models used here assumed that each antibody response was stimulated by a single stage of the schistosome life cycle and targeted a single stage. In reality, overlapping antigen expression between different stages [57] means that antibody responses to a specific antigen could be stimulated by more than one stage of the life cycle and could have damaging effects upon multiple life stages. The models described here could easily be extended to test this, although the number of potential combinations to be tested would vastly increase. A more feasible first step might be to model responses to particular antigens for which the antigen-specific antibody responses have been measured and the stage-specific expression is known, such as Sm22.6 [58].

These results suggest that gradual accumulation of exposure to protective antigens is sufficient to explain the slow development of protective immunity, without it being necessary to invoke a major role for dying worms or an antigen threshold, but do suggest that high levels of exposure to the relevant antigen throughout life are most likely to induce such a protective response. These models have suggested that protective immunity could primarily target worm fecundity, which was not routinely considered in earlier immuno-epidemiological models. They have also shown that the assumed worm survival curve can make a considerable difference to the outcome, challenging the usual assumption of exponential worm survival, which is mathematically convenient but has not been empirically demonstrated. This analysis has highlighted the importance of the combination of the stage of the life-cycle stimulating and targeted by protective antibody responses in determining age infection and antibody profiles, and has excluded a large number of potential combinations as being incompatible with field data. We have shown that pattern-oriented modelling can be a useful approach to use to distinguish between different hypotheses and conceptual models for immune development in human schistosome infection.

## Supporting Information

Figure S1 Memory decay rates for memory models which pass all criteria. Plots show the total number of times parameter combinations including the different possible combinations of decay rates for the two memory cell populations pass all criteria. (A,B) Models without cross-regulation or thresholds, (C,D) cross-regulation models (total frequencies summed over all of them), (E,F) models with a threshold on *M*_2_. The left-hand panels are for models with exponentially-distributed worm life span (*n* = 1) (A,C,E), the right-hand ones are for models with approximately Gaussian-distributed worm life span (*n* = 9) (B,D,F). All of the different combinations of decay rates that were used have a bar on the chart; black bars indicate that no successful parameter combination had this combination of memory decay rates, blue bars that at least one successful parameter combination had this combination of memory decay rates. Note that different maximum values are used on the *z* (frequency)-axis.
(TIF)

Figure S2 Mean natural worm life span for plasma cell models which pass all criteria. Plots show the total number of times parameter combinations including the different possible values for natural worm life span are able to meet all criteria for plasma cell models, with results broken down by the life cycle stage providing the antigenic stimulus for *A*_2_. Yellow bars: mean natural life span 3 years; light green bars: mean natural worm lifespan 6.5 years; dark green bars: mean natural worm lifespan 10 years. (A,B) Plasma cell models without cross-regulation or thresholds, (C,D) total frequencies summed over all of the cross-regulation models and (E,F) models with a threshold on *A*_2_. The left-hand panels are for models with exponentially-distributed worm life span (*n* = 1) (A,C,E), the right-hand ones are for models with approximately Gaussian-distributed worm life span (*n* = 9) (B,D,F). Note that different maximum values are used on the *y*-axis.
(TIF)

Figure S3 Mean natural worm life span for memory models which pass all criteria. See legend for figure S2.
(TIF)

## References

[1] Gryseels B, Polman K, Clerinx J, Kestens L (2006). Human schistosomiasis.. Lancet.

[2] van der Werf MJ, de Vlas SJ, Brooker S, Looman CWN, Nagelkerke NJD (2003). Quantification of clinical morbidity associated with schistosome infection in sub-Saharan Africa.. Acta Trop.

[3] Jordan P, Webbe G, Sturrock RF (1993). Human Schistosomiasis.

[4] Mott KE (1983). A reusable polyamide filter for diagnosis of *S. hematobium* infection by urine filtration.. Bull Soc Pathol Exot.

[5] Fulford AJC, Butterworth AE, Ouma JH, Sturrock RF (1995). A statistical approach to schistosome population-dynamics and estimation of the life-span of *Schistosoma mansoni* in man.. Parasitology.

[6] Wilkins HA, Goll PH, de C. Marshall TF, Moore PJ (1984). Dynamics of *Schistosoma haematobium* infection in a Gambian community. III. Acquisition and loss of infection.. Trans R Soc Trop Med Hyg.

[7] Clarke V de V (1966). The influence of acquired resistance in the epidemiology of bilharziasis.. Cent Afr J Med.

[8] Wilkins HA, Blumenthal UJ, Hagan P, Hayes RJ, Tulloch S (1987). Resistance to reinfection after treatment of urinary schistosomiasis.. Trans R Soc Trop Med Hyg.

[9] Chandiwana SK, Taylor P, Clarke VD (1988). Prevalence and intensity of schistosomiasis in 2 rural areas in Zimbabwe and their relationship to village location and snail infection rates.. Ann Trop Med Parasit.

[10] Woolhouse MEJ (1998). Patterns in parasite epidemiology: the peak shift.. Parasitol Today.

[11] Woolhouse MEJ (1992). A theoretical framework for the immunoepidemiology of helminth infection.. Parasite Immunol.

[12] Mutapi F, Ndhlovu PD, Hagan P, Woolhouse MEJ (1997). A comparison of humoral responses to *Schistosoma haematobium* in areas with low and high levels of infection.. Parasite Immunol.

[13] Ndhlovu P, Cadman H, Vennervald BJ, Christensen NO, Chidimu M (1996). Age-related antibody profiles in *Schistosoma haematobium* infections in a rural community in Zimbabwe.. Parasite Immunol.

[14] Hagan P, Blumenthal UJ, Dunn D, Simpson AJ, Wilkins HA (1991). Human IgE, IgG4 and resistance to reinfection with *Schistosoma haematobium*.. Nature.

[15] Woolhouse MEJ, Mutapi F, Ndhlovu PD, Chandiwana SK, Hagan P (2000). Exposure, infection and immune responses to *Schistosoma haematobium* in young children.. Parasitology.

[16] Mutapi F, Ndhlovu PD, Hagan P, Spicer JT, Mduluza T (1998). Chemotherapy accelerates the development of acquired immune responses to *Schistosoma haematobium* infection.. J Infect Dis.

[17] Woolhouse MEJ, Hagan P (1999). Seeking the ghost of worms past.. Nat Med.

[18] Mutapi F, Burchmore R, Mduluza T, Midzi N, Turner CMR (2008). Age-related and infection intensity-related shifts in antibody recognition of defined protein antigens in a schistosome-exposed population.. J Infect Dis.

[19] Anderson RM, May RM (1985). Herd-immunity to helminth infection and implications for parasite control.. Nature.

[20] Mitchell KM, Mutapi F, Woolhouse MEJ (2008). The predicted impact of immunosuppression upon population age-intensity profiles for schistosomiasis.. Parasite Immunol.

[21] Fulford AJC, Butterworth AE, Sturrock RF, Ouma JH (1992). On the use of age-intensity data to detect immunity to parasitic infections, with special reference to *Schistosoma mansoni* in Kenya.. Parasitology.

[22] Duerr HP, Dietz K, Eichner M (2003). On the interpretation of age-intensity profiles and dispersion patterns in parasitological surveys.. Parasitology.

[23] Grimm V, Revilla E, Berger U, Jeltsch F, Mooij WM (2005). Pattern-oriented modeling of agent-based complex systems: lessons from ecology.. Science.

[24] Wiegand K, Saltz D, Ward D, Levin SA (2008). The role of size inequality in self-thinning: a pattern-oriented simulation model for arid savannas.. Ecol Model.

[25] Swanack TM, Grant WE, Forstner MRJ (2009). Projecting population trends of endangered amphibian species in the face of uncertainty: a pattern-oriented approach.. Ecol Model.

[26] Rossmanith E, Blaum N, Grimm V, Jeltsch F (2007). Pattern-oriented modelling for estimating unknown pre-breeding survival rates: the case of the Lesser Spotted Woodpecker (*Picoides minor*).. Biol Conserv.

[27] Jenkins SJ, Hewitson JP, Jenkins GR, Mountford AP (2005). Modulation of the host's immune response by schistosome larvae.. Parasite Immunol.

[28] Pearce EJ (2005). Priming of the immune response by schistosome eggs.. Parasite Immunol.

[29] Skelly PJ, Wilson RA (2006). Making sense of the schistosome surface.. Adv Parasit.

[30] Smithers SR, Terry RJ, Hockley DJ (1969). Host antigens in schistosomiasis.. Proc R Soc Lond B Biol Sci.

[31] Abbas AK, Murphy KM, Sher A (1996). Functional diversity of helper T lymphocytes.. Nature.

[32] Jeannin P, Lecoanet S, Delneste Y, Gauchat J-F, Bonnefoy J-Y (1998). IgE versus IgG4 production can be differentially regulated by IL-10.. J Immunol.

[33] Woolhouse MEJ, Taylor P, Matanhire D, Chandiwana SK (1991). Acquired immunity and epidemiology of *Schistosoma haematobium*.. Nature.

[34] Goddard MJ, Jordan P (1980). On the longevity of *Schistosoma mansoni* in man on St. Lucia, West Indies.. Trans R Soc Trop Med Hyg.

[35] Vermund SH, Bradley DJ, Ruiztiben E (1983). Survival of *Schistosoma mansoni* in the human host - estimates from a community-based prospective study in Puerto Rico.. Am J Trop Med Hyg.

[36] Milner T, Reilly L, Nausch N, Midzi N, Mduluza T (2010). Circulating cytokine levels and antibody responses to human *Schistosoma haematobium*: IL-5 and IL-10 levels depend upon age and infection status.. Parasite Immunol.

[37] Mutapi F, Winborn G, Midzi N, Taylor M, Mduluza T (2007). Cytokine responses to *Schistosoma haematobium* in a Zimbabwean population: contrasting profiles for IFN-γ, IL-4, IL-5 and IL-10 with age.. BMC Infect Dis.

[38] Mutapi F, Rujeni N, Bourke C, Mitchell KM, Nausch N (2011). *Schistosoma haematobium* treatment in 1–5 year old children: safety and efficacy of the antihelminthic drug praziquantel.. PLoS Negl Trop Dis.

[39] Chan MS, Mutapi F, Woolhouse MEJ, Isham VS (2000). Stochastic simulation and the detection of immunity to schistosome infections.. Parasitology.

[40] Woolhouse MEJ (1993). A theoretical framework for immune responses and predisposition to helminth infection.. Parasite Immunol.

[41] Wilson JN, Nokes DJ (1999). Do we need 3 doses of hepatitis B vaccine?. Vaccine.

[42] Wilson JN, Nokes DJ, Medley GF, Shouval D (2007). Mathematical model of the antibody response to hepatitis B vaccines: Implications for reduced schedules.. Vaccine.

[43] Morell A, Terry WD, Waldmann TA (1970). Metabolic properties of IgG subclasses in man.. J Clin Invest.

[44] Waldmann TA, Strober W (1969). Metabolism of immunoglobulins.. Prog Allergy.

[45] Woolhouse MEJ (1994). A theoretical framework for the immunoepidemiology of blocking antibodies to helminth infection.. Parasite Immunol.

[46] Press W, Teukolsky S, Vetterling W, Flannery B (2002). Numerical recipes in C++: the art of scientific computing.

[47] Pickles M, Foss AM, Vickerman P, Deering K, Verma S (2010). Interim modelling analysis to validate reported increases in condom use and assess HIV infections averted among female sex workers and clients in southern India following a targeted HIV prevention programme.. Sex Transm Infect.

[48] Gray D (2002). A role for antigen in the maintenance of immunological memory.. Nat Rev Immunol.

[49] Kalia V, Sarkar S, Gourley TS, Rouse BT, Ahmed R (2006). Differentiation of memory B and T cells.. Curr Opin Immunol.

[50] Ochsenbein AF, Pinschewer DD, Sierro S, Horvath E, Hengartner H (2000). Protective long-term antibody memory by antigen-driven and T help-dependent differentiation of long-lived memory B cells to short-lived plasma cells independent of secondary lymphoid organs.. Proc Natl Acad Sci U S A.

[51] Gambhir M, Basáñez M-G, Turner F, Kumaresan J, Grassly NC (2007). Trachoma: transmission, infection, and control.. Lancet Infect Dis.

[52] Radbruch A, Muehlinghaus G, Luger EO, Inamine A, Smith KGC (2006). Competence and competition: the challenge of becoming a long-lived plasma cell.. Nat Rev Immunol.

[53] Amanna IJ, Carlson NE, Slifka MK (2007). Duration of humoral immunity to common viral and vaccine antigens.. N Engl J Med.

[54] Chandiwana SK (1987). Community water-contact patterns and the transmission of *Schistosoma haematobium* in the highveld region of Zimbabwe.. Soc Sci Med.

[55] Chandiwana SK, Woolhouse ME (1991). Heterogeneities in water contact patterns and the epidemiology of *Schistosoma haematobium*.. Parasitology.

[56] Chan MS, Isham VS (1998). A stochastic model of schistosomiasis immuno-epidemiology.. Math Biosci.

[57] Curwen RS, Ashton PD, Johnston DA, Wilson RA (2004). The *Schistosoma mansoni* soluble proteome: a comparison across four life-cycle stages.. Mol Biochem Parasit.

[58] Fitzsimmons CM, McBeath R, Joseph S, Jones FM, Walter K (2007). Factors affecting human IgE and IgG responses to allergen-like *Schistosoma mansoni* antigens: molecular structure and patterns of *in vivo* exposure.. Int Arch Allergy Imm.

[59] Macallan DC, Wallace DL, Zhang Y, Ghattas H, Asquith B (2005). B-cell kinetics in humans: rapid turnover of peripheral blood memory cells.. Blood.

[60] Bradley DJ, McCullough FS (1973). Egg output stability and the epidemiology of *Schistosoma haematobium* Part II. An analysis of the epidemiology of endemic *S. haematobium*.. Trans R Soc Trop Med Hyg.

[61] Useh MF, Ejezie GC (1999). Modification of behaviour and attitude in the control of schistosomiasis. 1. Observations on water-contact patterns and perception of infection.. Ann Trop Med Parasitol.

[62] Agnew A, Fulford AJ, Mwanje MT, Gachuhi K, Gutsmann V (1996). Age-dependent reduction of schistosome fecundity in *Schistosoma haematobium* but not *Schistosoma mansoni* infections in humans.. Am J Trop Med Hyg.

[63] King CH, Muchiri EM, Ouma JH (1992). Age-targeted chemotherapy for control of urinary schistosomiasis in endemic populations.. Mem Inst Oswaldo Cruz.

