# Supplementary figures and images for: Explaining Observed Infection and Antibody Age-Profiles in Populations with Urogenital Schistosomiasis
Source: PLoS Comput Biol. 2011 Oct 20;7(10):e1002237. doi: 10.1371/journal.pcbi.1002237 (PMC3197645; doi:10.1371/journal.pcbi.1002237)

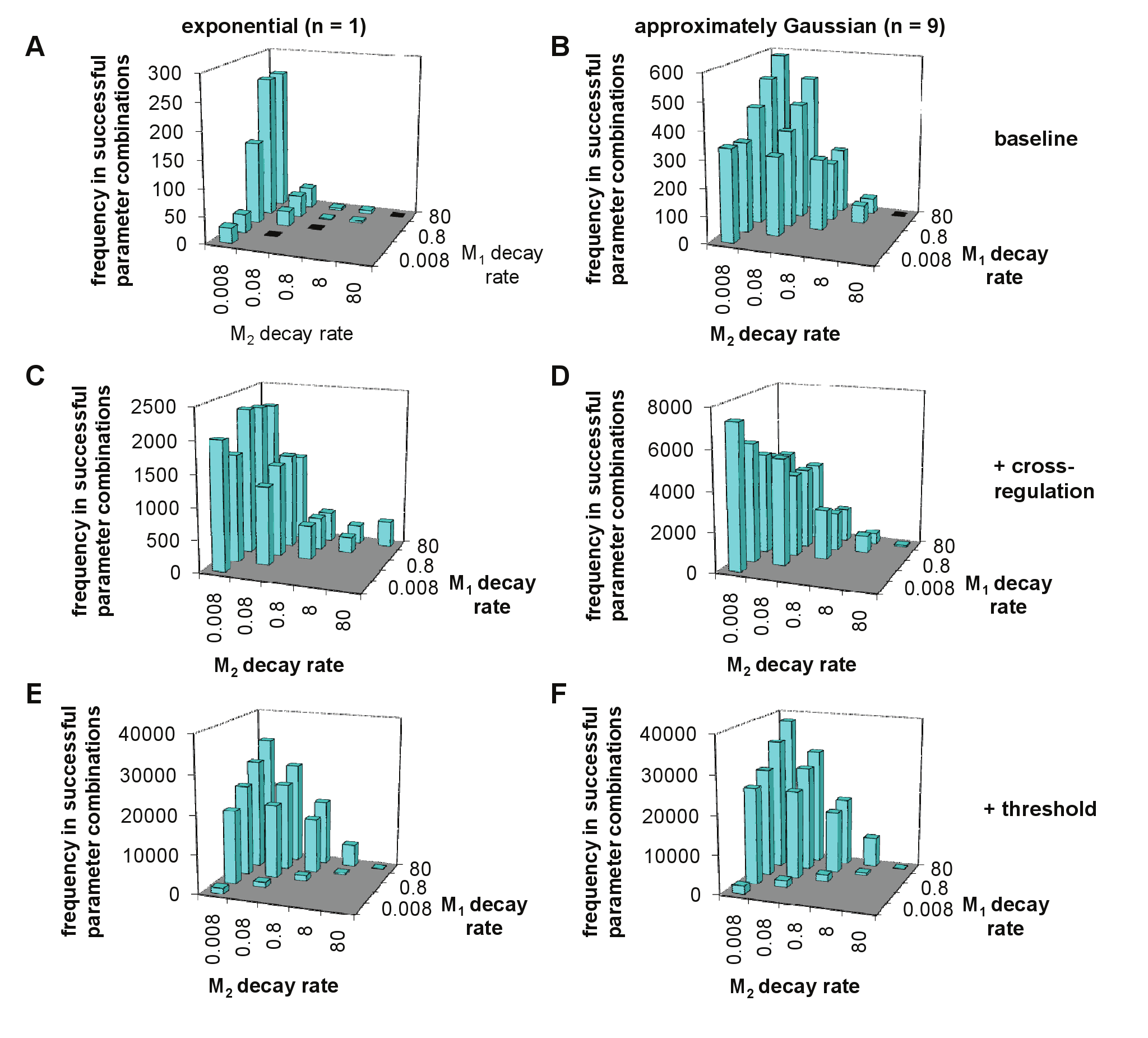

Supplement: Figure S1 — Memory decay rates for memory models which pass all criteria. Plots show the total number of times parameter combinations including the different possible combinations of decay rates for the two memory cell populations pass all criteria. (A,B) Models without cross-regulation or thresholds, (C,D) cross-regulation models (total frequencies summed over all of them), (E,F) models with a threshold on M 2. The left-hand panels are for models with exponentially-distributed worm life span (n = 1) (A,C,E), the right-hand ones are for models with approximately Gaussian-distributed worm life span (n = 9) (B,D,F). All of the different combinations of decay rates that were used have a bar on the chart; black bars indicate that no successful parameter combination had this combination of memory decay rates, blue bars that at least one successful parameter combination had this combination of memory decay rates. Note that different maximum values are used on the z (frequency)-axis. (TIF) [file pcbi.1002237.s001.tif]

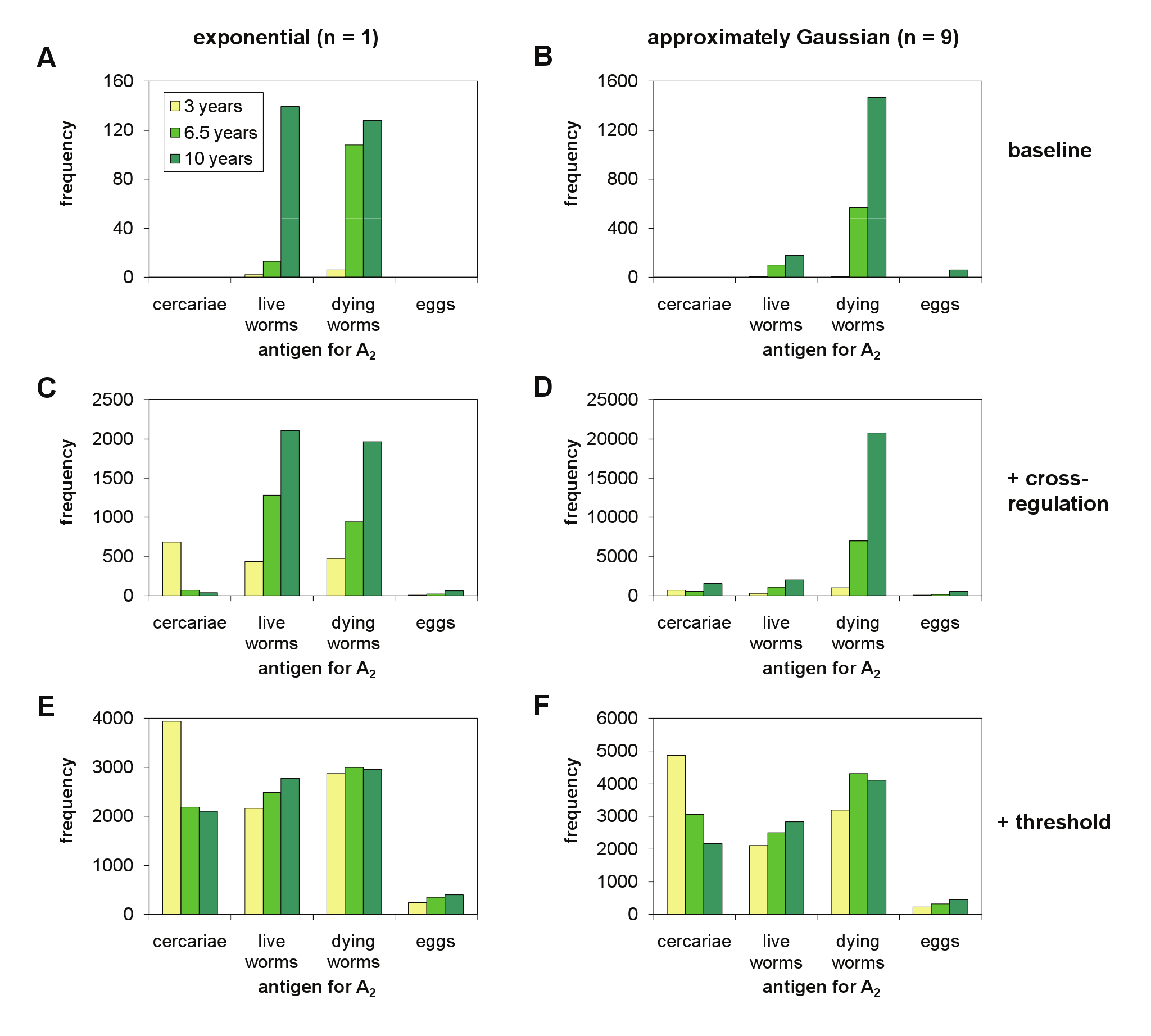

Supplement: Figure S2 — Mean natural worm life span for plasma cell models which pass all criteria. Plots show the total number of times parameter combinations including the different possible values for natural worm life span are able to meet all criteria for plasma cell models, with results broken down by the life cycle stage providing the antigenic stimulus for A 2. Yellow bars: mean natural life span 3 years; light green bars: mean natural worm lifespan 6.5 years; dark green bars: mean natural worm lifespan 10 years. (A,B) Plasma cell models without cross-regulation or thresholds, (C,D) total frequencies summed over all of the cross-regulation models and (E,F) models with a threshold on A 2. The left-hand panels are for models with exponentially-distributed worm life span (n = 1) (A,C,E), the right-hand ones are for models with approximately Gaussian-distributed worm life span (n = 9) (B,D,F). Note that different maximum values are used on the y-axis. (TIF) [file pcbi.1002237.s002.tif]

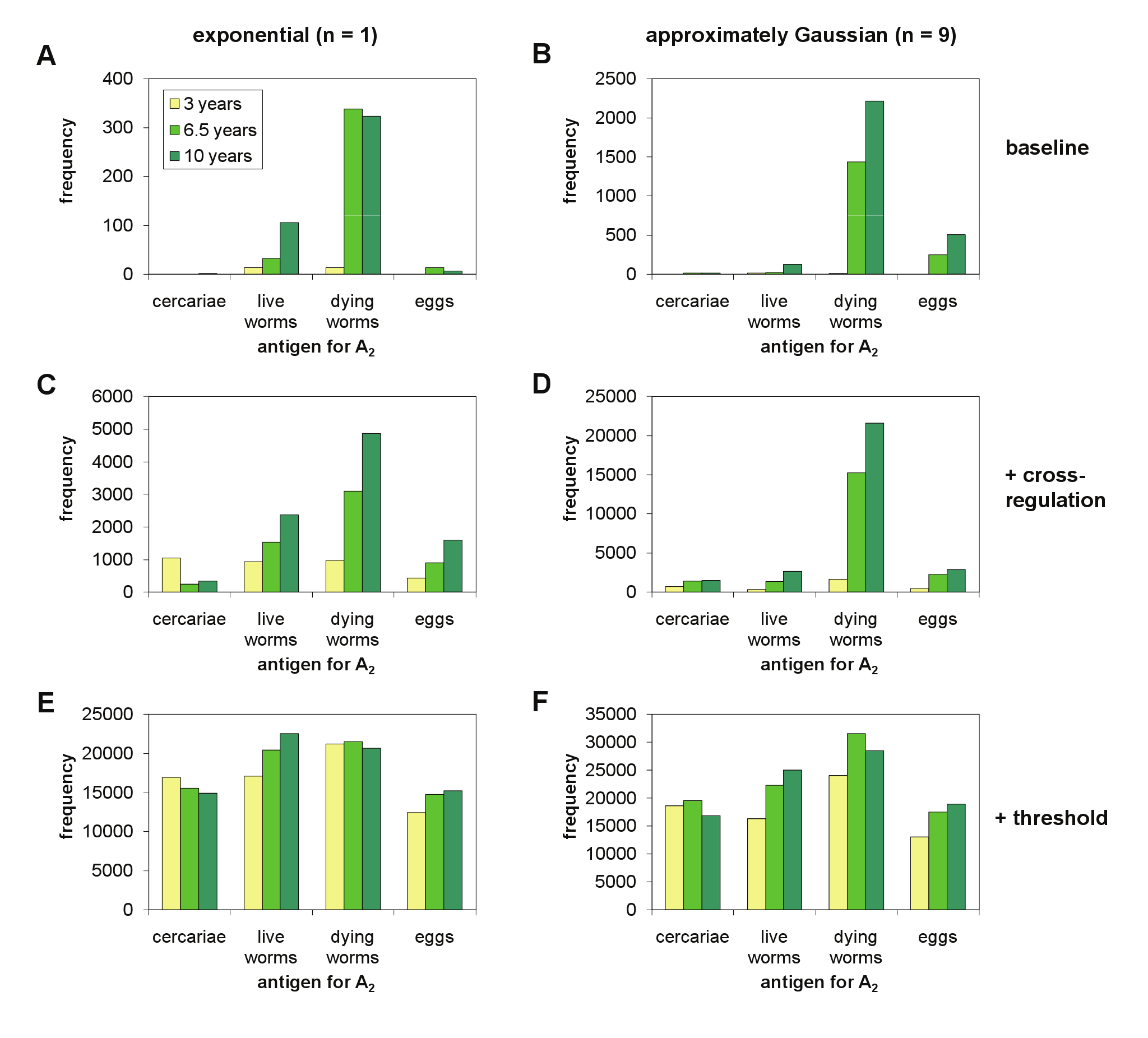

Supplement: Figure S3 — Mean natural worm life span for memory models which pass all criteria. See legend for figure S2. (TIF) [file pcbi.1002237.s003.tif]
